# Regular medical checkup program (in K-MEDI hub) to enhance the welfare of laboratory dogs and pigs

**DOI:** 10.1186/s42826-023-00170-7

**Published:** 2023-10-24

**Authors:** Gwang-Hoon Lee, Woori Jo, Joon-Suk Park, Tae-Ku Kang, Soo-Eun Sung, Taeho Oh, KilSoo Kim

**Affiliations:** 1 Preclinical Research Center, Daegu-Gyeongbuk Medical Innovation Foundation, Daegu, 41061 Republic of Korea; 2 Department of Veterinary Internal Medicine, College of Veterinary Medicine, Kyungpook National University, Daegu, 41566 Republic of Korea; 3 Department of Veterinary Toxicology, College of Veterinary Medicine, Kyungpook National University, Daegu, 41566 Republic of Korea

**Keywords:** Attending Veterinarian, Laboratory animal, Dog, Pig, Animal welfare, Medical checks

## Abstract

**Background:**

The importance of animal welfare is being recognized worldwide. Recently, the increasing demand for enhanced laboratory animal welfare has led to clinically featured transformations of animal research institutes. This study aims to describe the process and findings of veterinary medical check-ups and its influence on laboratory dogs and pigs welfare. Regular medical checkups were conducted by the attending veterinarian twice a year to ensure the health and welfare of dogs and pigs in our animal research institute. Based on the findings from the medical checkup, we assessed the current health of dogs and pigs,providing reasonable treatments to prevent the risk of complications.

**Results:**

Blood tests and physical examinations revealed clinically relevant findings. Some of these findings were due to insufficient postoperative care after invasive surgical experiments and the remaining were predictable side effects after surgical experiments. However, one finding involved severe gum bleeding due to retained deciduous teeth. This animal was euthanized because it was judged to reach the humane endpoint. Majority of the dogs and pigs at our animal research institute were considered to be healthy, based on the comprehensive results of the medical checkups.

**Conclusions:**

Regular medical checkups by the attending veterinarian established enhanced animal welfare, ensuring the accuracy and reproducibility of animal studies. This pioneering veterinary animal care program can serve as a potential advanced guideline for animal research institutes to improve dogs and pigs welfare.

**Supplementary Information:**

The online version contains supplementary material available at 10.1186/s42826-023-00170-7.

## Background

With progress in the healthcare industry and advanced technologies for the development of new drugs or medical devices, the number of animal research institutes have also increased worldwide. Animal research aims to provide scientific evidence prior to early feasible testing in humans and human clinical trials to assure the safety and efficacy of novel medical device technologies and therapies for humans and animals [1]. A well-designed animal model is a powerful tool to bridge the gap between basic scientific research and human clinical trials, called the “valley of death” due to the high risk of failure [2].

Laboratory animals, once considered only a biological reagent, are now being raised in animal research institutes, owing to the emerging global importance of ethical considerations [3, 4].

The welfare of laboratory animals in South Korea is regulated by the ANIMAL PROTECTION ACT with the principles of the 3Rs (Replacement, Reduction, and Refinement). The law emphasizes the importance of animal health by stating that “Animal testing shall be conducted in consideration of the enhancement of welfare of humankind and the dignity of lives of animals”. In addition, all animal research institutes should configure an Institutional Animal Care and Use Committee (IACUC) to ensure lawful compliance with the 3Rs [5].

Since physiological indicators of laboratory animals vary depending on their environmental and health conditions, psychological stress or physical distress is considered as a potential trigger to substantial scientific and humane implications for the use of laboratory animals [6]. The standardization of animals forms the basis for animal studies; in particular, animal health conditions affect the reproducibility and accuracy of the animal study findings. Hence, high quality clinical care is required in terms of animal welfare [7, 8]. With the growing interest in animal protection, the importance of laboratory animal welfare has also gained importance recently [9]. Furthermore, it is essential for animal researchers to recognize stress as an important factor which can potentially affect experimental results. Well-educated professionals skilled to handle animal stress should be enrolled in animal research institutes [10].

Attending veterinarians are regular employees in laboratory animal facilities and are generally familiar with all the laboratory animal species. They play a significant role in the accreditation of the International Association for the Control of Laboratory Animals (AAALAC-i) or the Korean Excellent Laboratory Animal Facility (KELAF) by the Ministry of Food and Drug Safety. In particular, attending veterinarians play an important role in ensuring the health and a conducive environment for the laboratory animals, which can minimize unexpected variables in well-designed animal studies [11].

This study aimed to design and evaluate a method for improving laboratory dogs and pigs welfare through regular health screening programs conducted by attending veterinarians in an animal research institute. This program is expected to play a pioneering role in laboratory dogs and pigs welfare, which is being strengthened worldwide.

## Results

### Health checkup schedule

**Fig. 1:**
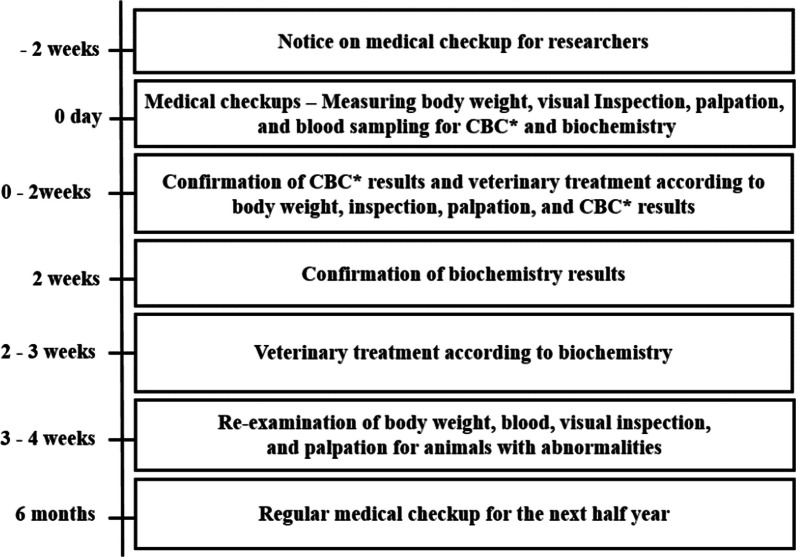
Schedule for semi-annually medical checkups of PRC, K-MEDI hub. *CBC: complete blood count

Health checkup of the animals was performed according to K-MEDI hub's health checkup procedure until euthanasia at the end of the experiment (Fig. 1). Researchers who wanted to apply for postponement or exclusion of medical checkups filled out the form and submitted it to attending veterinarian (Additional file 1: Fig. S1). During the medical checkup, individual health was recorded on the individual medical record form (Additional file 1: Fig. S2).

### Identification of clinical features and treatment for dogs

**Fig. 2:**
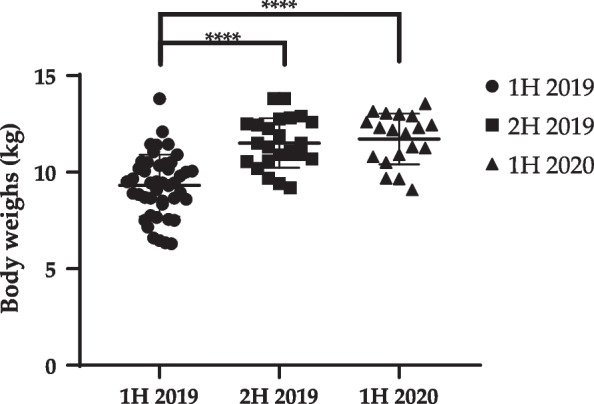
Change of body weight of dogs from 1H 2019 to 1H 2020. (****p < 0.0001 compared to 1H 2019)

**Fig. 3:**
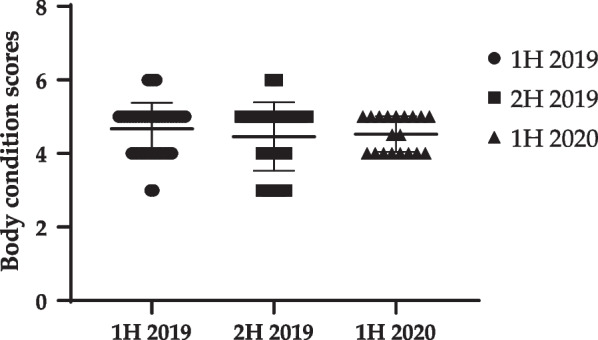
Change of body condition scores of dogs from 1H 2019 to 1H 2020

In first half of 2019 (1H 2019), the feed amount of dog-#29 increased from 150 g/day to 200 g/day due a body condition score (BCS) of 3/9. This BCS was 4/9 at the medical checkup in the second half of the year. Dog-#39 showed difficulty in feeding due to right upper deciduous canine tooth with bleeding gums. Considering animal welfare, this dog was euthanized with the early termination of experiments, to alleviate pain. In second half of 2019 (2H 2019), the BCSs of dog-#1 and dog-#28 were 6/9. To lose body weight, their feed amounts were decreased from 300 to 250 g. In contrast, since the BCS of dog-#22 was 3/9, the feed amount was increased from 200 to 300 g. Consequently, the BCSs of all the three dogs were 4/9 at the medical checkup in the second half of the year. Although the body weights significantly increased in 2H 2019 (11.51 ± 1.28) and first half of 2020 (1H 2020) (11.72 ± 1.32), compared to those in 1H 2019 (9.31 ± 1.61) (Table 1, Fig. 2), the average BCSs among the groups was similar, with no statistical significance (1H 2019, 4.67 ± 0.71/9; 2H 2019, 4.46 ± 0.93/9; and 1H 2020, 4.53 ± 0.49/9) (Fig. 3).

**Table 1:** Body weight, body condition score (BCS), and clinical features of dogs

Animal number	1H 2019				2H 2019				1H 2020		
	Body weight (kg)	Body temperature (°C)	BCS	Clinical feature	Body weight (kg)	Body temperature (°C)	BCS	Clinical feature	Body weight (kg)	Body temperature (°C)	BCS
Dog-#1	12.1	37.8	5	ND	13.8	10.9	3	Feed amount 200 g → 300 g ^2^	12.3	38.9	4
Dog-#2	8.5	38.5	5	ND	9.2	38.1	5	ND	9.65	39	4.5
Dog-#3	8.35	38.5	5	ND	Euthanized						
Dog-#4	11.4	38.7	5	ND	Euthanized						
Dog-#5	7.75	39.3	4	ND	Euthanized						
Dog-#6	7.15	39.3	4	ND	Euthanized						
Dog-#7	8.65	38.8	5	ND	Euthanized						
Dog-#8	9.1	39	5	ND	Euthanized						
Dog-#9	8.95	38.6	4	ND	Euthanized						
Dog-#10	9.45	39.3	6	ND	9.42	38.9	3	ND	10.9	38.8	4
Dog-#11	9.3	37.4	4	ND	Euthanized						
Dog-#12	9.65	38.1	5	ND	Euthanized						
Dog-#13	10.5	38.2	5	ND	Euthanized						
Dog-#14	10.05	38.8	5	ND	Euthanized						
Dog-#15	10.5	37.6	4	ND	9.7	38.2	3	Monocytosis^3^	13.55	38.9	5
Dog-#16	10.45	38.3	5	ND	10.9	38.5	4	Mild labored breathing^4^, neutrophilia ^5^	12	39.6	4
Dog-#17	9.8	38.1	5	ND	10.9	38.1	4	ND	10.8	39.2	4
Dog-#18	10.55	39	5	ND	12.6	38	5	ND	13.15	39.2	5
Dog-#19	10.9	38.8	5	ND	12.45	37.8	5	ND	12.45	38.6	4
Dog-#20	13.8	37.6	5	ND	Euthanized						
Dog-#21	11.45	38.7	5	ND	13.8	39.3	5	ND	12.6	40.2	5
Dog-#22	11.05	38.7	5	ND	11.5	38.8	3	Feed amount 200 g → 300 g	12.9	39	4
Dog-#23	9.5	38.6	5	ND	10.2	39.1	5	ND	Euthanized		
Dog-#24	11.45	38.4	6	ND	12.75	39.5	3	ND	13	39.6	5
Dog-#25	10.35	38.5	5	ND	10.9	41.1	5	Mild labored breathing^4^	12.3	40	5
Dog-#26	10.15	38.6	6	ND	12.9	38.6	4	ND	Euthanized		
Dog-#27	7.65	39	4	ND	Euthanized						
Dog-#28	10	38.5	5	ND	12.8	38.4	6	Feed amount 300 g → 200 g	13.05	39.1	4
Dog-#29	7.5	38.6	3	Feed amount: 150 g → 200 g	Euthanized						
Dog-#30	10.05	38.3	5	ND	11.9	38.1	5	Mild labored breathing^4^	12.2	39.5	5
Dog-#31	6.3	39.3	4	ND	Euthanized						
Dog-#32	8.65	38.6	5	ND	10.7	38.7	5	ND	10.5	38.9	4
Dog-#33	9.45	37.6	5	ND	11.3	38.9	4	ND			
Dog-#34	8.6	39.3	5	ND	Euthanized						
Dog-#35	6.6	39.3	4	ND	Euthanized						
Dog-#36	10.2	38	4	ND	10.6	38.5	6	ND	11.3	39.1	5
Dog-#37	8.85	38.4	6	ND	11.5	38.8	4	ND	11.25	39.1	5
Dog-#38	9.45	37.6	4	ND	11.1	38.5	5	The right hind leg lameness Monocytosis^5^	Euthanized		
Dog-#39	6.45	38.8	3	Retained deciduous teeth of right upper canine, bleeding^1^	Euthanized by humane endpoint						
Dog-#40	6.35	37.1	4	ND	Euthanized						
Dog-#41	7.5	38.3	4	ND	Euthanized						
Dog-#42	7.55	37.6	4	ND	Euthanized						
Dog-#43	8.7	38.6	4	ND	10.55	38.5	5	Neutrophila^6^	Euthanized		
Dog-#44	8.9	37.6	4	ND	Euthanized						
Dog-#45	9.5	38.8	5	ND	Euthanized						
Dog-#46	Before bringing in animals				12.25	39.1	5	ND	Euthanized		
Dog-#47					12.5	39.1	5	ND	9.7	39	5
Dog-#48					Before bringing in animals				9.1	38.9	4.5

ND: Not detected; Euthanized: Euthanized due to study termination; BCS: Body condition score. Superscript numbers indicate the actions taken, as follows:

^1^Difficulty in feeding due to right upper deciduous canine tooth with bleeding gums → euthanizing with early termination of experiments to alleviate pain, considering animal welfare

^2^BCS was 3/9 → feed for dog-#29 increased from 150 g/day to 200 g/day. The BCS of the dog was 4/9 at medical checkup during the second half.

^3^Monocytosis after a bronchial transplant experiment, detected 2 weeks before examination → antibiotics treatment (enrofloxacin 5 mg/kg once daily (SID) for 3 days). Blood re-analysis after 3 weeks—The monocyte concentration in blood decreased to 1.81 × 10^3^ cells/uL

^4^Mild labored breathing 2 weeks after bronchial transplant experiment → breathing of the dogs normalized after 3 weeks and maintained during a medical examination in 1H 2020

^5^Lameness of the right hind leg was found in dog-#16 with monocyte after the right hind leg implant experiment 1 week before the medical checkup → antibiotics treatment (enrofloxacin 5 mg/kg SID for 3 days). Blood re-analysis in 3 weeks—Monocyte normalized to 0.83 × 10^3^ cells/uL, the dog did not show lameness after 3 weeks

^6^neutrophilia after bronchial transplant experiments → antibiotics treatment (enrofloxacin 5 mg/kg SID for 3 days). Blood re-analysis after 3 weeks—Neutrophil count normalized to 6.50 × 10^3^ cells/uL

Further, dog-#15 showed monocytosis after a bronchial transplant experiment, two weeks before examination. This dog was treated with enrofloxacin 5 mg/kg once daily (SID) for 3 days; on re-examination after 3 weeks, the blood monocyte concentration (×10^3^ cells/uL) decreased to 1.81 × 10^3^ cells/uL, which was within the normal range (Additional file 2: Tables 3 and 4).

Dog-#16, dog-#25, and dog-#30 showed mild labored breathing 2 weeks after a bronchial transplant experiment, and dog-#16 had neutrophilia. However, breathing in the dogs normalized after 3 weeks and the blood neutrophil concentration of dog-#16 normalized to 7.87 × 10^3^ cells/uL. All the three dogs showed normal levels at the medical check in 1H 2020. One week before the medical checkup, dog-#16 showed lameness in the right hind leg with monocytosis after an implant experiment. After treatment with enrofloxacin 5 mg/kg SID for 3 days, the blood monocyte concentration normalized to 0.83 × 10^3^ cells/uL, and the dog did not show lameness after 3 weeks (Additional file 2: Tables 1 and 2).

Dog-#43 showed neutrophilia after bronchial transplant experiments. After enrofloxacin 5 mg/kg SID for 3 days, the blood neutrophil concentration normalized to 6.50 × 10^3^ cells/uL. In 1H 2020, no clinical features were found in any of the dogs (Table 1, Additional file 2: Tables 5 and 6).

### Identification of clinical features and treatment for pigs

**Fig. 4:**
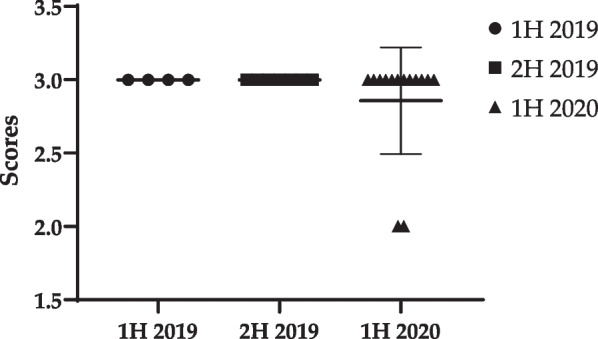
Change of body condition scores of pigs from 1H 2019 to 1H 2020

In 1H 2020, the BCSs of pig-#14 and pig-#23 were 2/5. To regain normal body weight, the feed amount was increased from 500 to 700 g and the feed was mixed with sugar, to increase palatability. In 1H 2019 and 2H 2019, no clinical feature was found (Table 2). All the BCSs were 3/5, except for 2/5 among pigs in 1H 2020. Therefore, the average BCSs were similar among the groups, with statistically significant difference (1H 2019: 3.00 ± 0.00/5, 2H 2019: 3.00 ± 0.00/5, and 1H 2020: 2.86 ± 0.36/5) (Fig. 4).

**Table 2:** The BCSs of pigs

Animal number	1H 2019		2H 2019		1H of 2020	
	Body weight (kg)	BCS	Body weight (kg)	BCS	Body weight (kg)	BCS
Pig-#1	37.2	3	Euthanized			
Pig-#2	37.5	3	Euthanized			
Pig-#3	37.1	3	Euthanized			
Pig-#4	37.3	3	Euthanized			
Pig-#5	Before bringing in animals		37.8	3	Euthanized	
Pig-#6	Before bringing in animals		38.5	3	Euthanized	
Pig-#7	Before bringing in animals		37.5	3	Euthanized	
Pig-#8	Before bringing in animals		37.5	3	Euthanized	
Pig-#9	Before bringing in animals		37.6	3	Euthanized	
Pig-#10	Before bringing in animals		38.2	3	37.9	3
Pig-#11	Before bringing in animals		37.5	3	37.8	3
Pig-#12	Before bringing in animals				38.1	3
Pig-#13	Before bringing in animals				39.2	3
Pig-#14	Before bringing in animals				37.8	2^1^
Pig-#15	Before bringing in animals				38.5	3
Pig-#16	Before bringing in animals				37.8	3
Pig-#17	Before bringing in animals				36.9	3
Pig-#18	Before bringing in animals				37.7	3
Pig-#19	Before bringing in animals				37.8	3
Pig-#20	Before bringing in animals				37.4	3
Pig-#21	Before bringing in animals				36.6	3
Pig-#22	Before bringing in animals				37.4	3
Pig-#23	Before bringing in animals				37.4	2^1^
Pig-#24	Before bringing in animals				Euthanized	
Pig-#25	Before bringing in animals				Euthanized	

Euthanized: Euthanized due to study termination; BCS: Body condition score

^1^After checking the BCS to be 2/5, sugar water was added to the feed to increase feed preference, along with an increase in feed

## Discussion

Laboratory animals are used for biological research or educational purposes; the number of laboratory animals used in accordance with the developing bio-industry is also increasing [12]. In Korea, the number of laboratory animals used was 183.4 million in 2012, 28.7 million in 2016, and 371.2 million in 2019, rising to an annual average of 14.6% between 2012 and 2019. As social interest in animal welfare has increased, the UK farm animal welfare council has established five freedoms for animals to improve animal welfare in 1979 and the laws to protect animals have been implemented globally [13]. Welfare of laboratory animals is also being improved to address inescapable stress factors such as experimental routines, invasive experiment, unwanted companion, transportation, and handling [6, 14].

Veterinarians are employed not only in animal clinical care facilities in animal hospitals but also in various fields such as food sanitation and safety, protection from biological hazards, defense against common infectious diseases, conservation of biodiversity, and animal protection. Among these, the importance of veterinarians in laboratory animal facilities has been emphasized recently [15]. Veterinarians in animal research institutes are responsible for the health and welfare of laboratory animals and are termed as attending veterinarians (AV). They have access to all the animals in the facility, to provide veterinary care [15].

Laboratory animals are commonly defined as live vertebrates produced for or used for the purpose of scientific research, testing, or teaching. Preclinical animal research refers to the testing of a candidate new drug, procedure, or other medical treatment in laboratory animals prior to human clinical trials [15].

The Ministry of Food and Drug Safety in Korea has designated mice, rats, hamsters, gerbil, guinea pigs, rabbits, dogs, pigs, and monkeys as priority laboratory animals. Rodents, which are relatively small animals, including mice and rats, are preferred for evaluating new drug efficacy due to cost-effectiveness, short life-span, and easy handling [16]. In particular, compared to the characteristics of rodents, those of large animals including rabbits, dogs, pigs, or monkeys are anatomically, pathologically, and physiologically more similar to humans. Hence, many studies use large animals such as dogs or pigs [17]. Animal study institutions primarily ensure the accuracy and reproducibility of animal studies by achieving the normalization of laboratory animals [7, 8]. Quarantine is a process that involves restricting the movement of animals, animal products, vectors, and fomites that have been exposed or infected with disease, in order to prevent any direct or indirect contact with the native animal population for a specific duration [18]. As part of that effort, quarantine process is implemented for animals in the animal research facility. In K-MEDI hub, the AV conducts a comprehensive examination and physical assessment of the dogs and pigs upon arrival, searching for any indications of injury or illness. Additionally, blood samples and other specimens can be collected for laboratory testing to identify any potential diseases or infections under the judgment of the AV according to their clinical signs, with the AV utilizing their clinical expertise to interpret the results. The quarantine period lasts for a duration of two weeks, during which the AV monitors the animals for clinical symptoms and analyzes the laboratory results. Upon completion of the quarantine period, the AV determines the end of quarantine for each individual animal.

In this study, we conducted regular medical checkups on dogs and pigs twice a year, based on which appropriate measures could be taken to improve the welfare and standardization of the laboratory animals. Blood tests, including complete blood cell count (CBC) and blood biochemistry were performed after aseptic blood collection. In addition, BCSs were measured and specifications were recorded through auscultation, visual inspection, and palpation. BCS is a verified method of scoring animals by physical inspection [19, 20]; the ideal BCSs are 4 or 5 out of 9 for dogs and 3 out of 5 for pigs [21, 22]. During the 1.5 year health check-up period, majority of the clinical symptoms for dogs and pigs in our facility were mild.

The BCSs of 3/9 or 6/9 in 4 dogs were normalized to 4/9 or 5/9 by adjusting the feed amount. Optimal body weight is essential for the health and welfare of animals, since BCS ≥ 6/9 (overweight) or BCS ≤ 3/9 (underweight) may be associated with disease or could serve as a predictor of a clinical condition [23]. Dogs with a BCS ≥ 6/9 should have reduced-energy content maintenance diets for modest weight reduction, since being overweight is commonly associated with morbidities such as diabetes, metabolic diseases, or cardiovascular diseases [24–26]. Therefore, we attempted to normalize the body weight of animals by controlling the amount of feed.

The reason BCS was unaffected despite weight gain among dogs in 2H 2019, compared to that in 1H 2019 could be attributed to age-dependent musculoskeletal growth. All the dogs in the present study were beagles, who continue to grow upto 18 months of age [27]. The average ages of dogs in 1H 2019 and 2H 2019 were 14 and 19 months, respectively. Thus, the discrepancy in BCSs and body weight was reasonable.

Moreover, ideal BCSs (4/9 or 5/9) were observed among all dogs in 1H 2020, contrary to those among 1H 2019 and 2H 2019 dogs. Therefore, the S.D value of 1H 2020 (4.67 ± 0.71) was smaller than that of 1H 2019 (5.46 ± 0.93) and 2H 2019 (4.53 ± 0.49). Although only one pig in 1H 2020 showed a BCS 2/5, there was no additional clinical symptom. We inferred that this was due to successful regular calibration of BCSs with control of amount of feed and veterinary treatment, including antibiotic use to treat clinical symptoms such as monocytosis and neutrophilia, which can be induced during animal experiments. Therefore, regular health checkups by the AV are imperative to derive reliable results, since because factors causing distress can affect the reproducibility and reliability of animal studies [28].

Despite the commitment to ensure the ideal health of laboratory animals, there are limitations relative to companion animals. First, the dog with bleeding gums due to retained deciduous teeth was euthanized, considering the principle of ‘refinement’ among the 3Rs, since the dog was in severe pain, accompanied by excessive bleeding. Contrary to companion animals, laboratory animals have a wider “humane endpoint”—the scientifically justified point for pain or distress [29]. The human endpoint for companion animals is guided by human trials while that in laboratory animals relies on emerging safety concerns or the judgement that pain or distress cannot be alleviated through treatment. [15, 29]. We judged that tooth extraction for treatment caused extreme pain, and bleeding gums due to retained deciduous teeth met humane euthanasia standards. Second, there was lack of continuous prolonged study data for pigs, since all the three medical checkups were not performed for any of the pigs. Third, data reproducibility could not be confirmed in this study because breeding animals with different ages, sexes, and origins were investigated.

Contrary to companion animals, laboratory animals are raised in limited space and are exposed to invasive surgical experiments. It is a methodological challenge for AV to overcome the detrimental environments. Therefore, further studies on regular medical checkups should evaluate the psychological satisfaction of animals according to the provided environmental enrichment and post-operative pain control methods.

We introduced highly enhanced animal welfare methods, the history of regular medical checkup established in this study will develop a roadmap for improving welfare and standardization of laboratory dogs and pigs in animal research institute.

## Conclusions

In conclusion, this study introduced enhanced animal welfare methods; the study design of regular medical checkups established in this study will aid in developing a roadmap for improving the welfare of laboratory dogs and pigs in an animal research institute.

## Methods

### Animal research institute

K-MEDI hub is a high-tech medical industry cluster created to strategically cater to the global new drug and medical device industries and supports the optimization of new drug candidates, design of medical devices, prototyping, and efficacy evaluation. K-MEDI hub consists of centers for new drug development, medical device development, preclinical research, and clinical drug manufacturing. Among these, the preclinical research center (PRC), which harbors a breeding system for mice, rats, rabbits, dogs, pigs, and monkey is specialized for laboratory animal research such as efficacy/pre-biological stability assessment of new drugs and the performance of new medical devices.

The PRC, K-MEDI hub, was certified by the Ministry of Food and Drug Safety in Korea as KELAF in 2016 and fully accredited by the Association for Assessment and Accreditation of Laboratory Animal Care International (AAALAC-i) in 2020. AAALAC-i certification is recognized as an organization for non-clinical studies that encourages the humane treatment of animals in scientific research and ensures optimum animal care and use [30]. These certifications are awarded only if there is an attending veterinarian (AV) who is responsible for the clinical care of animals and welfare of laboratory animals in the institute.

The capacities for mice, rats, rabbits, dogs, pigs, and monkey at PRC were 18,000, 1800, 100, 45, 35, and 46 respectively. The K-MEDI hub conducted preclinical evaluations by importing approximately 10,000 mice, 3000 rats, 250 rabbits, 50 dogs, and 40 pigs annually. Moreover, in 2023, they aim to address unmet needs for non-human primate preclinical research by importing roughly 20 non-human primates.

Rodent breeding rooms were maintained as specific pathogen free barrier colonies established by the AV and experts in microbiological monitoring. Large animals such as rabbits, dogs, and pigs were reared separately from rodents to prevent cross infection. The IACUC of K-MEDI hub reviews whether exploring alternatives, rationale for the proposed number of animals, minimizing animal discomfort, and humane endpoints are included in the protocol. K-MEDI hub employs one AV who is responsible for animal quarantine, veterinary management, post-approval monitoring, and serving as a member of the Institutional Animal Care and Use Committee (IACUC), with a critical role in ensuring the ethical use of animals in research and maintaining high standards of animal welfare.

The post-approval monitoring process of IACUC with the AV semi-annually ensures that the study is being conducted in accordance with the approval and that the researchers protect animal welfare.

In this study, to enhance the animal welfare for laboratory animals, PRC, K-MEDI hub itself conducted regular medical check-up programs for laboratory animals, including dogs and pigs, by the AV to ensure the health and welfare of animals and perform appropriate veterinary treatments and environmental management in the facilities.

### Animals

A medical examination was conducted for all the dogs and pigs in breeding. Dogs and pigs were selected as the target species, since blood collection was possible without sacrifice and research involves a relatively long study period, contrary to that in rodents.

In 1H 2019, 45 dogs (41 males, 4 females) and 4 pigs (1 male, 3 females) were examined, and in 2H 2019, 24 dogs (21 males, 7 females) and 7 pigs (7 females) were examined; 21 dogs (21 males) and 14 pigs (14 females) were examined in 1H 2020.

### Blood test

Blood collected using a sterilized syringe was placed into a serum-separating tube (SST) and an ethylenediaminetetraacetic acid tube and separated from serum in the SST tube.

White blood cells (WBC), red blood cells, hemoglobin, hematocrit, mean corpuscular volume, mean corpuscular hemoglobin, mean corpuscular hemoglobin concentration in blood, red blood cell distribution width, platelets, and WBC differential count were analyzed as CBC using a hematology system with an autoslide (ADVIA 2120i, Siemens, WA, USA).

The levels of aspartate aminotransferase, albumin, alanine aminotransferase, total bilirubin, triglyceride, blood urea nitrogen, calcium, creatinine, inorganic phosphorus, glucose, sodium, total cholesterol, potassium, total protein, chloride, and C-reactive protein were analyzed as blood biochemistry using a clinical chemistry analyzer (TBA-120FR, Toshiba, Tokyo, Japan).

### Clinical symptoms

Along with visual inspection, the body weight, auscultation, and palpation were measured along with determining the BCSs of dogs and pigs. Dogs are scored out of 9 and pigs are scored out of 5 [21, 22]. Dogs are categorized based on scoring the following points: 1,2, and 3-too thin; 4 and 5-ideal; 6-above ideal; 7-overweight; 8 and 9-obese. Pigs are categorized based on scoring the following points: 1-emaciated, 2-thin, 3-ideal, 4-fat, and 5-overly fat.

### Statistical analyses

Statistical significance was determined using GraphPad Prism 8 (GraphPad Software Inc., San Diego, CA, USA). All data are presented as mean ± standard deviation. In case of changes in the body weight of dogs, the normality test was passed and one-way analysis of variance with Tukey's multiple comparisons test was used. P < 0.05 was considered as statistically significant. In case of changes in the BCSs of dogs and the body weights of pigs, normality tests were not passed, and Kruskal–Wallis tests were used.

### Supplementary Information

**Additional file 1**. **Supplementary Figure 1**. Medical checkup postponement / exemption application form of K-MEDI hub. **Supplementary Figure 2**. Individual medical record form of K-MEDI hub.

**Additional file 2**. **Supplementary Table 1**. The results for complete blood cell count in 1H 2019. **Supplementary Table 2**. The results for blood biochemistry in 1H 2019. **Supplementary Table 3**. The results for complete blood cell count in 2H 2019. **Supplementary Table 4**. The results for blood biochemistry in 2H 2019. **Supplementary Table 5**. The results for complete blood cell count in 1H 2020. **Supplementary Table 6**. The results for blood biochemistry in 1H 2020.

## Data Availability

The datasets during and/or analysed during the current study available from the corresponding author on reasonable request.

## Abbreviations

AAALAC-i: International association for the control of laboratory animals
BCS: Body condition score
CBC: Complete blood cell count
IACUC: Institutional animal care and use committee
KELAF: Korean excellent laboratory animal facility
PRC: Preclinical research center
SST: Serum-separating tube
WBC: White blood cells
