# Supplementary material for: Regular medical checkup program (in K-MEDI hub) to enhance the welfare of laboratory dogs and pigs
Source: Lab Anim Res. 2023 Oct 24;39:24. doi: 10.1186/s42826-023-00170-7 (PMC10594746; doi:10.1186/s42826-023-00170-7)
Supplement: Supplementary file 1 — Additional file 1. Supplementary Figure 1. Medical checkup postponement / exemption application form of K-MEDI hub. Supplementary Figure 2. Individual medical record form of K-MEDI hub. [file 42826_2023_170_MOESM1_ESM.docx]

***Additional File 1***

**
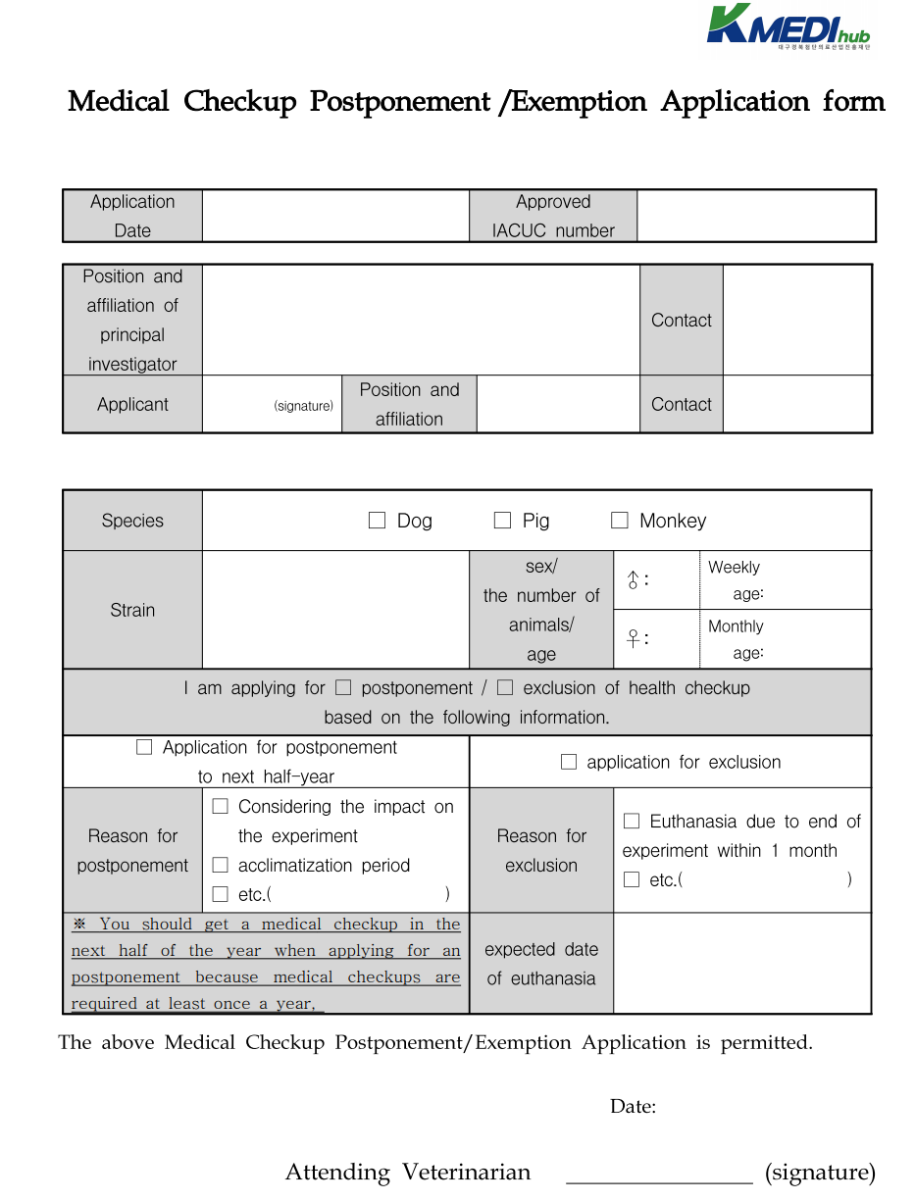
**

**Supplementary Figure 1.** Medical checkup postponement / exemption application form of K-MEDI hub


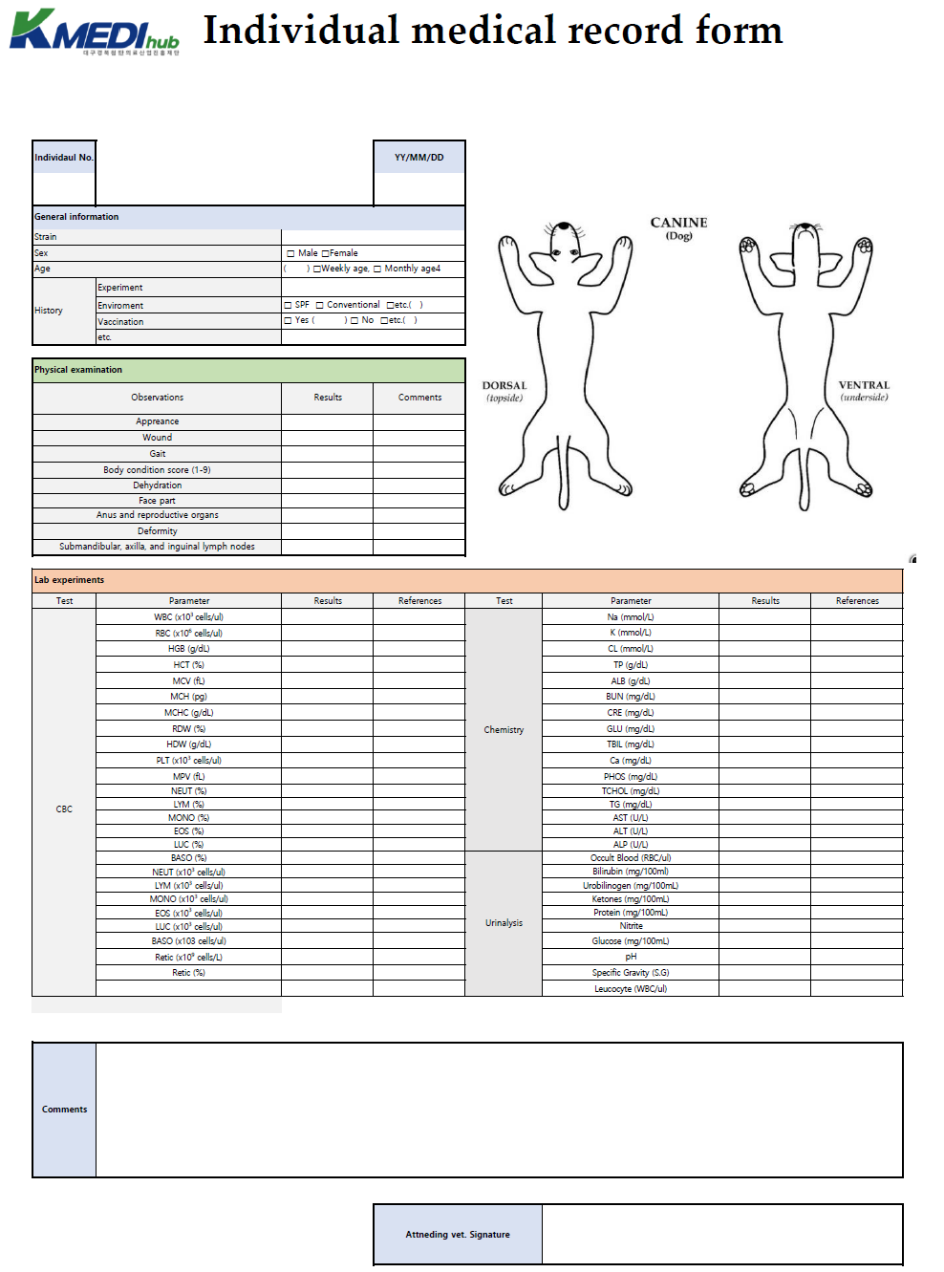


**Supplementary Figure 2.** Individual medical record form of K-MEDI hub
