# Supplementary material for: Regular medical checkup program (in K-MEDI hub) to enhance the welfare of laboratory dogs and pigs
Source: Lab Anim Res. 2023 Oct 24;39:24. doi: 10.1186/s42826-023-00170-7 (PMC10594746; doi:10.1186/s42826-023-00170-7)
Supplement: Supplementary file 2 — Additional file 2. Supplementary Table 1. The results for complete blood cell count in 1H 2019. Supplementary Table 2. The results for blood biochemistry in 1H 2019. Supplementary Table 3. The results for complete blood cell count in 2H 2019. Supplementary Table 4. The results for blood biochemistry in 2H 2019. Supplementary Table 5. The results for complete blood cell count in 1H 2020. Supplementary Table 6. The results for blood biochemistry in 1H 2020. [file 42826_2023_170_MOESM2_ESM.docx]

***Additional File 2***

**Supplementary Table 1**. The results for complete blood cell count in 1H 2019

| **Sample Name** | WBC  (×10^3 cells/µL) | RBC  (×10^6 cells/µL) | HGB  (g/dL) | HCT  (%) | MCV  (fL) | MCH  (pg) | MCHC  (g/dL) | RDW  (%) | HDW  (g/dL) | PLT  (×10^3 cells/uL) | MPV  (fL) | NEUT  (%) | LYM  (%) | MONO  (%) | EOS  (%) | LUC  (%) | BASO  (%) | NEUT  (×10^3 cells/µL) | LYM  (×10^3 cells/µL) | MONO  (×10^3 cells/µL) | EOS  (×10^3 cells/µL) | LUC  (×10^3 cells/µL) | BASO  (×10^3 cells/µL) | Retic  (×10^9 cells/L) | Retic  (%) |
| --- | --- | --- | --- | --- | --- | --- | --- | --- | --- | --- | --- | --- | --- | --- | --- | --- | --- | --- | --- | --- | --- | --- | --- | --- | --- |
| Dog-#1 | 12.66 | 6.89 | 15.1 | 41.1 | 59.6 | 21.9 | 36.7 | 13.9 | 3.97 | 448 | 7.9 | 57.4 | 30.1 | 7 | 4.6 | 0.2 | 0.7 | 7.27 | 3.81 | 0.89 | 0.58 | 0.03 | 0.08 | 75 | 1.09 |
| Dog-#2 | 8.76 | 8.25 | 18.5 | 56.8 | 68.8 | 22.4 | 32.5 | 11.9 | 1.78 | 445 | 9.3 | 66.1 | 25.3 | 3.9 | 3.8 | 0.3 | 0.5 | 5.79 | 2.22 | 0.34 | 0.34 | 0.02 | 0.04 | 82.9 | 1 |
| Dog-#3 | 10.05 | 7.42 | 16.5 | 50.9 | 68.5 | 22.2 | 32.4 | 13 | 1.8 | 425 | 8.8 | 62.9 | 29.2 | 5.5 | 1.9 | 0.2 | 0.3 | 6.32 | 2.93 | 0.56 | 0.2 | 0.02 | 0.03 | 96.5 | 1.3 |
| Dog-#4 | 9.48 | 7.11 | 16 | 49.4 | 69.5 | 22.6 | 32.5 | 11.8 | 1.79 | 390 | 9.7 | 52.3 | 28.9 | 9.5 | 8.5 | 0.4 | 0.4 | 4.95 | 2.74 | 0.9 | 0.81 | 0.04 | 0.04 | 107.4 | 1.51 |
| Dog-#5 | 7.24 | 6.43 | 15.3 | 40.5 | 63 | 23.8 | 37.8 | 19.3 | 6.62 | 392 | 10 | 59.7 | 28.6 | 7.9 | 2.2 | 0.4 | 1.3 | 4.32 | 2.07 | 0.57 | 0.16 | 0.03 | 0.09 | 51.8 | 0.81 |
| Dog-#6 | 6.35 | 6.51 | 14.5 | 41.5 | 63.7 | 22.3 | 35 | 15 | 3.93 | 284 | 9.7 | 60.3 | 26.6 | 8.9 | 3.4 | 0.3 | 0.6 | 3.83 | 1.69 | 0.56 | 0.21 | 0.02 | 0.04 | 26.6 | 0.41 |
| Dog-#7 | 7.94 | 7.22 | 15.7 | 42.2 | 58.5 | 21.8 | 37.3 | 17 | 5.13 | 386 | 9.6 | 49.4 | 40.8 | 6.3 | 1.7 | 0.3 | 1.5 | 3.92 | 3.24 | 0.5 | 0.14 | 0.03 | 0.12 | 68.4 | 0.95 |
| Dog-#8 | 7.56 | 7.55 | 17.1 | 45.7 | 60.5 | 22.7 | 37.5 | 18 | 5.87 | 438 | 8.6 | 54.3 | 31.5 | 4.9 | 8.4 | 0.2 | 0.7 | 4.1 | 2.38 | 0.37 | 0.63 | 0.02 | 0.05 | 78.4 | 1.04 |
| Dog-#9 | 8.46 | 7.2 | 17.1 | 45.5 | 63.3 | 23.7 | 37.5 | 16.7 | 5.24 | 346 | 8.2 | 64.2 | 23.5 | 5.7 | 5.8 | 0 | 0.8 | 5.44 | 1.99 | 0.48 | 0.49 | 0 | 0.06 | 60.2 | 0.84 |
| Dog-#10 | 7.26 | 8.92 | 17.5 | 56.2 | 63 | 19.6 | 31.1 | 12.3 | 1.9 | 521 | 8.6 | 38.9 | 42.3 | 11.5 | 6.2 | 0.7 | 0.3 | 2.82 | 3.07 | 0.83 | 0.45 | 0.05 | 0.05 | 120.2 | 1.35 |
| Dog-#11 | 9.18 | 6.76 | 14.7 | 42.2 | 62.4 | 21.7 | 34.8 | 13.1 | 4.22 | 373 | 8.9 | 55.8 | 33.4 | 8.2 | 1.7 | 0.4 | 0.6 | 5.12 | 3.06 | 0.75 | 0.16 | 0.03 | 0.05 | 35.8 | 0.53 |
| Dog-#12 | 10.32 | 6.75 | 14.8 | 42.9 | 63.6 | 21.9 | 34.4 | 14.3 | 4.11 | 438 | 9.7 | 58.3 | 28.7 | 7.9 | 4.2 | 0.3 | 0.6 | 6.02 | 2.96 | 0.82 | 0.43 | 0.03 | 0.06 | 80.1 | 1.19 |
| Dog-#13 | 9.29 | 6.45 | 15.3 | 43.6 | 67.5 | 23.7 | 35.2 | 12.8 | 3.15 | 214 | 10.2 | 63.2 | 27.6 | 6.6 | 2.1 | 0.1 | 0.4 | 5.87 | 2.56 | 0.61 | 0.2 | 0.01 | 0.04 | 91.7 | 1.42 |
| Dog-#14 | 6.01 | 6.81 | 14.8 | 47.4 | 69.6 | 21.7 | 31.2 | 12.5 | 1.9 | 163 | 10.5 | 57.6 | 31.4 | 8 | 2.3 | 0.2 | 0.5 | 3.46 | 1.88 | 0.48 | 0.14 | 0.01 | 0.03 | 81.9 | 1.2 |
| Dog-#15 | 8.52 | 6.83 | 15.7 | 42.3 | 62 | 23 | 37.1 | 12.7 | 4.11 | 325 | 9.1 | 52.1 | 33.6 | 6.9 | 6.1 | 0.3 | 1 | 4.44 | 2.86 | 0.59 | 0.52 | 0.02 | 0.09 | 68.2 | 1 |
| Dog-#16 | 11.17 | 7.73 | 16.3 | 45.1 | 58.3 | 21.1 | 36.1 | 13.6 | 4.07 | 360 | 10.5 | 58.8 | 29.9 | 5.1 | 4.5 | 0.6 | 1.1 | 6.57 | 3.35 | 0.57 | 0.5 | 0.06 | 0.12 | 82.7 | 1.07 |
| Dog-#17 | 9.34 | 7.49 | 17.2 | 52.3 | 69.8 | 22.9 | 32.8 | 11.5 | 1.92 | 265 | 9.3 | 56.3 | 28.2 | 7.3 | 7.6 | 0.2 | 0.4 | 5.26 | 2.64 | 0.68 | 0.71 | 0.02 | 0.04 | 51.6 | 0.69 |
| Dog-#18 | 7.26 | 6.74 | 14.5 | 43.3 | 64.3 | 21.5 | 33.4 | 13.4 | 2.59 | 307 | 10.6 | 62.6 | 27.8 | 5.8 | 3.1 | 0.2 | 0.6 | 4.54 | 2.01 | 0.42 | 0.23 | 0.02 | 0.04 | 89.5 | 1.33 |
| Dog-#19 | 8.53 | 6.43 | 14.7 | 44 | 68.4 | 22.9 | 33.5 | 14 | 3.15 | 287 | 10.7 | 60.6 | 31.5 | 5.4 | 1.7 | 0.2 | 0.7 | 5.17 | 2.69 | 0.46 | 0.14 | 0.01 | 0.06 | 57.2 | 0.89 |
| Dog-#20 | 7.81 | 7.41 | 16.5 | 42.1 | 56.8 | 22.3 | 39.2 | 16.1 | 4.7 | 283 | 9.8 | 51.9 | 37.6 | 7.3 | 1.5 | 0.2 | 1.5 | 4.05 | 2.94 | 0.57 | 0.12 | 0.01 | 0.11 | 16.1 | 0.22 |
| Dog-#21 | 9.43 | 6.67 | 15.1 | 40.4 | 60.5 | 22.6 | 37.4 | 15.6 | 4.08 | 276 | 10.8 | 55.2 | 33.7 | 8.4 | 2 | 0.2 | 0.5 | 5.21 | 3.18 | 0.79 | 0.19 | 0.02 | 0.05 | 48.8 | 0.73 |
| Dog-#22 | 10.52 | 6.16 | 13.6 | 34.7 | 56.4 | 22 | 39.1 | 18.4 | 4.78 | 609 | 7.7 | 54.6 | 32.1 | 5.9 | 6.7 | 0.2 | 0.5 | 5.74 | 3.38 | 0.62 | 0.7 | 0.02 | 0.06 | 25.5 | 0.41 |
| Dog-#23 | 7.69 | 5.8 | 13.1 | 34.7 | 59.8 | 22.7 | 37.9 | 16.7 | 5.24 | 263 | 10.2 | 59.1 | 29.8 | 8.3 | 2.1 | 0.2 | 0.5 | 4.55 | 2.29 | 0.64 | 0.16 | 0.01 | 0.04 | 15.6 | 0.27 |
| Dog-#24 | 7.7 | 6.84 | 14.7 | 37.8 | 55.2 | 21.5 | 39 | 20 | 5.92 | 229 | 10.7 | 52.7 | 35.4 | 7.4 | 3.3 | 0.2 | 1 | 4.06 | 2.72 | 0.57 | 0.26 | 0.02 | 0.08 | 23.8 | 0.35 |
| Dog-#25 | 9.96 | 7.13 | 15 | 39 | 54.7 | 21 | 38.4 | 16.9 | 5.55 | 320 | 9.9 | 52.8 | 39.3 | 5.4 | 1.5 | 0.2 | 0.8 | 5.26 | 3.92 | 0.54 | 0.15 | 0.02 | 0.08 | 28.4 | 0.4 |
| Dog-#26 | 10.42 | 6.63 | 15 | 46.6 | 70.3 | 22.6 | 32.2 | 12.3 | 1.73 | 212 | 9.4 | 52.3 | 38.5 | 5.2 | 1.8 | 0.9 | 1.3 | 5.45 | 4.01 | 0.55 | 0.19 | 0.09 | 0.13 | 96.7 | 1.46 |
| Dog-#27 | 7.14 | 6.47 | 14.8 | 41.9 | 64.7 | 22.8 | 35.2 | 13.4 | 3.9 | 344 | 8.9 | 60.3 | 29 | 7.9 | 1.8 | 0.3 | 0.8 | 4.3 | 2.07 | 0.56 | 0.13 | 0.02 | 0.06 | 54.8 | 0.85 |
| Dog-#28 | 7.5 | 6.43 | 14.3 | 44.4 | 69.1 | 22.3 | 32.3 | 12.2 | 1.72 | 312 | 9.8 | 65.6 | 21.7 | 7.5 | 4.7 | 0.2 | 0.3 | 4.92 | 1.63 | 0.56 | 0.36 | 0.02 | 0.02 | 65.4 | 1.02 |
| Dog-#29 | 10.18 | 5.92 | 13.4 | 42.3 | 71.5 | 22.7 | 31.7 | 12.7 | 1.64 | 381 | 8.8 | 55.3 | 32.9 | 7.3 | 4 | 0.2 | 0.3 | 5.63 | 3.35 | 0.75 | 0.41 | 0.02 | 0.03 | 99.2 | 1.67 |
| Dog-#30 | 4.99 | 6.54 | 15.1 | 46.3 | 70.8 | 23 | 32.5 | 13.9 | 2.72 | 302 | 9.5 | 44.4 | 41.4 | 8.4 | 4.3 | 0.2 | 1.2 | 2.22 | 2.06 | 0.42 | 0.21 | 0.01 | 0.06 | 165.5 | 2.53 |
| Dog-#31 | 7.96 | 6.1 | 13.3 | 42.1 | 69 | 21.8 | 31.6 | 14.4 | 2.85 | 310 | 9.7 | 51.8 | 34 | 6.5 | 6.5 | 0.4 | 0.8 | 4.12 | 2.7 | 0.52 | 0.52 | 0.03 | 0.06 | 108.4 | 1.78 |
| Dog-#32 | 9.17 | 6.88 | 15.8 | 45.6 | 66.3 | 23 | 34.7 | 13 | 2.6 | 307 | 9.5 | 59.8 | 29.7 | 7.7 | 2.3 | 0.2 | 0.4 | 5.48 | 2.72 | 0.7 | 0.21 | 0.02 | 0.04 | 56.6 | 0.82 |
| Dog-#33 | 8.9 | 6.5 | 14.7 | 46.4 | 71.4 | 22.6 | 31.7 | 12.5 | 2.53 | 280 | 10.8 | 50.3 | 36.9 | 6.1 | 6.1 | 0.2 | 0.4 | 4.47 | 3.28 | 0.55 | 0.54 | 0.02 | 0.04 | 51.7 | 0.79 |
| Dog-#34 | 8.27 | 6.2 | 14.1 | 44.5 | 71.7 | 22.8 | 31.8 | 12.4 | 1.78 | 347 | 9.5 | 58.4 | 29.7 | 7.5 | 3.7 | 0.2 | 0.5 | 4.83 | 2.46 | 0.62 | 0.31 | 0.02 | 0.04 | 74.4 | 1.2 |
| Dog-#35 | 10.93 | 6.08 | 13 | 40.1 | 66 | 21.3 | 32.3 | 15.5 | 3.15 | 437 | 8.6 | 55.6 | 37 | 6.1 | 0.9 | 0.1 | 0.2 | 6.08 | 4.04 | 0.66 | 0.1 | 0.01 | 0.02 | 116.8 | 1.92 |
| Dog-#36 | 14.92 | 5.71 | 13.1 | 40.8 | 71.5 | 23 | 32.2 | 12.6 | 1.75 | 335 | 9.8 | 74.3 | 14.7 | 7 | 3.6 | 0.2 | 0.2 | 11.09 | 2.19 | 1.05 | 0.53 | 0.03 | 0.03 | 85.3 | 1.49 |
| Dog-#37 | 9.49 | 7.21 | 15.7 | 44.7 | 62 | 21.8 | 35.1 | 14.2 | 4.19 | 324 | 10.1 | 58.4 | 31.7 | 7.4 | 1.5 | 0.3 | 0.7 | 5.54 | 3.01 | 0.7 | 0.14 | 0.03 | 0.07 | 49.8 | 0.69 |
| Dog-#38 | 10.89 | 5.81 | 12.8 | 39.5 | 68 | 22.1 | 32.5 | 13.5 | 2.44 | 335 | 9.6 | 60.6 | 30.7 | 7.3 | 0.7 | 0.3 | 0.4 | 6.6 | 3.35 | 0.79 | 0.08 | 0.03 | 0.04 | 91.5 | 1.57 |
| Dog-#39 | 7.68 | 6.39 | 14 | 40.5 | 63.3 | 21.9 | 34.5 | 14.7 | 4.6 | 387 | 8.8 | 59.3 | 28.2 | 9.1 | 2.3 | 0.2 | 0.9 | 4.56 | 2.16 | 0.7 | 0.18 | 0.02 | 0.07 | 86.2 | 1.35 |
| Dog-#40 | 11.09 | 7 | 15.4 | 47.7 | 68.1 | 22 | 32.3 | 11.9 | 2.22 | 380 | 8.2 | 59.2 | 29 | 9.4 | 1.5 | 0.3 | 0.7 | 6.56 | 3.21 | 1.04 | 0.16 | 0.03 | 0.08 | 61.4 | 0.88 |
| Dog-#41 | 9.4 | 6.63 | 14.9 | 46.8 | 70.6 | 22.5 | 31.9 | 13.1 | 1.96 | 347 | 8.3 | 51.2 | 37.5 | 9.7 | 0.9 | 0.3 | 0.5 | 4.81 | 3.53 | 0.91 | 0.08 | 0.03 | 0.04 | 124.9 | 1.88 |
| Dog-#42 | 9.53 | 6.41 | 14.3 | 44.4 | 69.3 | 22.3 | 32.2 | 12.6 | 1.97 | 309 | 9.7 | 64.9 | 23.6 | 8.8 | 2.3 | 0.2 | 0.3 | 6.19 | 2.25 | 0.84 | 0.22 | 0.02 | 0.02 | 116.4 | 1.81 |
| Dog-#43 | 10.38 | 7.79 | 16.8 | 47.7 | 61.2 | 21.6 | 35.2 | 13.1 | 3.96 | 316 | 9.4 | 59.7 | 31.5 | 6.5 | 1.3 | 0.3 | 0.7 | 6.2 | 3.27 | 0.68 | 0.13 | 0.03 | 0.08 | 95.4 | 1.22 |
| Dog-#44 | 10.68 | 5.85 | 14.4 | 40 | 68.4 | 24.5 | 35.9 | 16.9 | 4.8 | 222 | 9.3 | 63 | 23.8 | 5 | 7.3 | 0.2 | 0.7 | 6.72 | 2.54 | 0.54 | 0.78 | 0.02 | 0.07 | 40 | 0.68 |
| Dog-#45 | 10.12 | 7.04 | 16 | 43.7 | 62.1 | 22.8 | 36.7 | 14.6 | 4.42 | 433 | 9.9 | 62.7 | 28.3 | 6.2 | 2 | 0.1 | 0.7 | 6.35 | 2.87 | 0.63 | 0.2 | 0.01 | 0.07 | 95.3 | 1.35 |
| Pig-#1 | 6.68 | 4.51 | 10.3 | 31.1 | 68.9 | 22.9 | 33.3 | 13.8 | 1.54 | 329 | 8.4 | 49.9 | 43.1 | 4.8 | 0.2 | 1.9 | 0.1 | 3.33 | 2.88 | 0.32 | 0.01 | 0.12 | 0 | 40.6 | 0.9 |
| Pig-#2 | 9.09 | 6.32 | 13.4 | 43.3 | 68.5 | 21.2 | 31 | 13.5 | 1.54 | 300 | 8 | 56 | 38.8 | 4 | 0.3 | 0.8 | 0.1 | 5.09 | 3.53 | 0.36 | 0.03 | 0.07 | 0.01 | 18.3 | 0.29 |
| Pig-#3 | 9.67 | 5.42 | 11.8 | 37.4 | 69.1 | 21.9 | 31.6 | 14.6 | 1.56 | 291 | 8.5 | 52.8 | 40.9 | 4.4 | 0.5 | 1.3 | 0.1 | 5.1 | 3.95 | 0.43 | 0.05 | 0.13 | 0.01 | 49 | 0.9 |
| Pig-#4 | 11.64 | 6.55 | 13.4 | 42.9 | 65.4 | 20.5 | 31.3 | 14.6 | 1.67 | 470 | 7 | 50.2 | 44.8 | 3.8 | 0.4 | 0.7 | 0.1 | 5.84 | 5.21 | 0.44 | 0.05 | 0.09 | 0.01 | 74.5 | 1.14 |

* WBC: White blood cell, RBC: Red blood cell, HGB: Hemoglobin, HCT: hematocrit, MCV: mean corpuscular volume, MCH: mean corpuscular hemoglobin, MCHC: mean corpuscular hemoglobin concentration, RDW: Red Blood Cell Distribution Width, HDW: hemoglobin distribution width, PLT: platelet, MPV: Mean platelet volume, NEUT: Neutrophil, LYM: Lymphocyte, MONO: Monocyte, EOS: Eosinophil, LUC: Large unstained cell, Retic: Reticulocyte.

**Supplementary Table 2**. The results for blood biochemistry in 1H 2019

| **Sample Name** | Na (mmol/L) | K (mmol/L) | CL (mmol/L) | TP (g/dL) | ALB (g/dL) | BUN  (mg/dL) | CRE  (mg/dL) | GLU  (mg/dL) | TBIL  (mg/dL) | Ca  (mg/dL) | PHOS  (mg/dL) | TCHOL (mg/dL) | TG (mg/dL) | AST  (U/L) | ALT (U/L) | CRP (mg/dL) |
| --- | --- | --- | --- | --- | --- | --- | --- | --- | --- | --- | --- | --- | --- | --- | --- | --- |
| Dog-#1 | 142 | 4.8 | 106.8 | 6.5 | 3.14 | 24 | 0.7 | 66 | 0 | 10.6 | 4.1 | 171 | 118 | 28 | 36 | 0 |
| Dog-#2 | 145.1 | 4.6 | 108.2 | 6.4 | 3.16 | 23.5 | 0.8 | 68 | 0 | 10.6 | 4.1 | 166 | 97 | 33 | 30 | 0 |
| Dog-#3 | 144.3 | 5.1 | 109.8 | 5.7 | 2.96 | 25.4 | 0.8 | 76 | 0 | 10.2 | 3.7 | 163 | 75 | 32 | 35 | 0.01 |
| Dog-#4 | 142.3 | 4.9 | 108.2 | 5.9 | 2.95 | 22.1 | 0.7 | 100 | 0 | 10.1 | 4.6 | 112 | 49 | 56 | 39 | 0 |
| Dog-#5 | 139.8 | 4.6 | 109.7 | 5.7 | 2.94 | 0 | 0.8 | 62 | 0.1 | 9.6 | 4 | 68 | 16 | 68 | 47 | 0.01 |
| Dog-#6 | 138.7 | 4.2 | 108.7 | 7.5 | 3.46 | 18.1 | 0.8 | 65 | 0.2 | 10.1 | 4.8 | 136 | 41 | 68 | 46 | 0.01 |
| Dog-#7 | 140.9 | 4.2 | 109.8 | 6.1 | 3.2 | 12.3 | 0.7 | 80 | 0 | 10.4 | 4.6 | 190 | 27 | 32 | 43 | 0.01 |
| Dog-#8 | 138 | 5 | 106.3 | 6.7 | 3.24 | 0 | 0.8 | 64 | 0.1 | 10.6 | 5.9 | 155 | 39 | 57 | 25 | 0.01 |
| Dog-#9 | 142.9 | 4.5 | 108.4 | 5.8 | 3.17 | 0 | 0.7 | 66 | 0 | 10.4 | 5 | 153 | 30 | 32 | 35 | 0.01 |
| Dog-#10 | 139.9 | 4.8 | 107.4 | 6.6 | 3 | 35.4 | 0.8 | 89 | 0 | 10.1 | 4.4 | 137 | 34 | 39 | 52 | 0.02 |
| Dog-#11 | 144.7 | 4 | 101.7 | 6.9 | 3.42 | 17.4 | 0.7 | 56 | 0.1 | 11.1 | 6 | 184 | 38 | 46 | 36 | 0.01 |
| Dog-#12 | 145.4 | 4.2 | 106 | 6.7 | 3.15 | 16.2 | 0.6 | 71 | 0 | 10.9 | 5.7 | 141 | 58 | 33 | 27 | 0.01 |
| Dog-#13 | 143.3 | 5.1 | 104.8 | 6.2 | 3.15 | 17.3 | 0.7 | 52 | 0 | 11.1 | 5.9 | 144 | 21 | 40 | 39 | 0 |
| Dog-#14 | 144.1 | 5.2 | 106.1 | 6.6 | 3.24 | 24.3 | 0.7 | 67 | 0 | 11.1 | 4.5 | 142 | 89 | 36 | 37 | 0 |
| Dog-#15 | 141.7 | 4.9 | 105.1 | 6 | 3.23 | 25.9 | 0.8 | 75 | 0 | 11 | 4.4 | 126 | 102 | 34 | 35 | 0 |
| Dog-#16 | 141.1 | 4.8 | 106.7 | 7.2 | 3.22 | 28.1 | 0.7 | 97 | 0 | 10.9 | 4.7 | 121 | 77 | 38 | 66 | 0.01 |
| Dog-#17 | 139.9 | 4.6 | 104.6 | 7 | 3.3 | 37.3 | 0.9 | 81 | 0.1 | 11.1 | 4.8 | 128 | 73 | 41 | 54 | 0.01 |
| Dog-#18 | 141.6 | 4.6 | 108 | 5.7 | 3.13 | 18.5 | 0.8 | 90 | 0 | 10.6 | 6.3 | 146 | 19 | 33 | 48 | 0 |
| Dog-#19 | 138.2 | 4.5 | 108.2 | 6.1 | 3.18 | 19.8 | 0.7 | 100 | 0.1 | 10.3 | 5.7 | 145 | 25 | 31 | 38 | 0.01 |
| Dog-#20 | 144.7 | 4.5 | 104.3 | 6.6 | 3.24 | 15.4 | 0.8 | 51 | 0.1 | 11.1 | 7 | 187 | 25 | 48 | 56 | 0.02 |
| Dog-#21 | 143.2 | 5 | 106.4 | 6.2 | 3.03 | 23.8 | 0.8 | 87 | 0 | 11 | 5.3 | 118 | 65 | 42 | 40 | 0 |
| Dog-#22 | 142.2 | 4.5 | 106.9 | 5.9 | 2.87 | 19 | 0.6 | 86 | 0 | 10.8 | 6 | 118 | 85 | 36 | 39 | 0 |
| Dog-#23 | 143.7 | 4.4 | 105.1 | 5.8 | 3.03 | 27.4 | 0.8 | 94 | 0 | 10.9 | 6 | 162 | 55 | 34 | 41 | 0.01 |
| Dog-#24 | 141.2 | 4.7 | 106.1 | 5.9 | 3.02 | 21.4 | 0.8 | 102 | 0 | 11 | 5.2 | 158 | 61 | 28 | 41 | 0 |
| Dog-#25 | 143.5 | 4.8 | 104.1 | 6.1 | 3.15 | 25.9 | 0.8 | 73 | 0 | 11.4 | 5.5 | 161 | 67 | 44 | 41 | 0 |
| Dog-#26 | 141.3 | 4.6 | 107.6 | 6.3 | 3.22 | 19.4 | 0.8 | 84 | 0.1 | 11.5 | 6.9 | 167 | 23 | 35 | 26 | 0 |
| Dog-#27 | 139.8 | 4.9 | 104.1 | 6.2 | 3.3 | 22.1 | 0.9 | 82 | 0.1 | 11.2 | 5.6 | 155 | 39 | 45 | 43 | 0.01 |
| Dog-#28 | 143 | 4.3 | 107.6 | 6 | 3.19 | 16.6 | 0.7 | 101 | 0 | 10.9 | 6.4 | 144 | 25 | 36 | 48 | 0 |
| Dog-#29 | 142.2 | 4.4 | 100.9 | 5.6 | 2.91 | 17.5 | 0.6 | 82 | 0 | 10.9 | 7.4 | 160 | 52 | 53 | 488 | 0.02 |
| Dog-#30 | 141.5 | 4.8 | 106.3 | 6.6 | 3.28 | 17 | 0.7 | 96 | 0.2 | 11.1 | 6.8 | 140 | 22 | 40 | 28 | 0.01 |
| Dog-#31 | 141.3 | 4.8 | 108.5 | 5.2 | 2.82 | 19.9 | 0.8 | 101 | 0 | 10.1 | 5 | 109 | 19 | 35 | 47 | 0 |
| Dog-#32 | 142.3 | 4.6 | 104.5 | 6.2 | 2.96 | 28.2 | 0.7 | 77 | 0 | 11.1 | 4.4 | 170 | 86 | 34 | 31 | 0.01 |
| Dog-#33 | 137.8 | 4.3 | 100 | 6.5 | 3.32 | 19.6 | 0.8 | 86 | 0.2 | 11.1 | 6.2 | 136 | 24 | 35 | 28 | 0.01 |
| Dog-#34 | 134.1 | 4.3 | 102.5 | 6.5 | 3.14 | 20.4 | 0.7 | 100 | 0.2 | 10.8 | 5.6 | 167 | 27 | 31 | 22 | 0.02 |
| Dog-#35 | 140.8 | 4.6 | 109.2 | 5.1 | 2.84 | 16.6 | 0.6 | 91 | 0 | 10.3 | 6.7 | 129 | 24 | 27 | 26 | 0 |
| Dog-#36 | 142.5 | 5 | 102.9 | 5.8 | 3.07 | 26.7 | 0.8 | 72 | 0 | 11.2 | 5.8 | 136 | 70 | 34 | 36 | 0.01 |
| Dog-#37 | 140.4 | 4.6 | 102.4 | 6.9 | 3.18 | 21.4 | 0.7 | 78 | 0.1 | 11.3 | 5.2 | 171 | 97 | 48 | 27 | 0 |
| Dog-#38 | 145.2 | 4.2 | 101.7 | 6.3 | 3.28 | 18.4 | 0.8 | 62 | 0.1 | 11.5 | 7.6 | 194 | 27 | 51 | 51 | 0 |
| Dog-#39 | 140.4 | 4.7 | 106 | 6.2 | 3.14 | 20.5 | 0.8 | 89 | 0.1 | 10.5 | 6 | 138 | 24 | 31 | 32 | 0 |
| Dog-#40 | 143.3 | 4.6 | 103 | 6.3 | 3.1 | 30.5 | 0.8 | 89 | 0 | 12.6 | 4.5 | 173 | 121 | 31 | 24 | 0 |
| Dog-#41 | 140.9 | 4.6 | 104.1 | 6 | 3.1 | 30.8 | 0.8 | 103 | 0 | 12 | 4.6 | 179 | 51 | 30 | 36 | 0.01 |
| Dog-#42 | 144 | 5 | 108.9 | 5.2 | 2.77 | 28.9 | 0.7 | 96 | 0 | 10.9 | 5.3 | 78 | 50 | 36 | 41 | 0 |
| Dog-#43 | 144.5 | 4.4 | 106 | 5.7 | 3.06 | 20.2 | 0.7 | 70 | 0 | 11.3 | 5.6 | 125 | 24 | 37 | 34 | 0.01 |
| Dog-#44 | 142.2 | 4.6 | 107.1 | 5.8 | 3 | 11.9 | 0.6 | 87 | 0 | 11.1 | 6.3 | 160 | 49 | 29 | 17 | 0 |
| Dog-#45 | 144.4 | 5 | 102.2 | 6.1 | 3.09 | 21.7 | 0.7 | 72 | 0 | 11.2 | 6 | 184 | 76 | 48 | 37 | 0 |
| Pig-#1 | 135.8 | 4.2 | 99.1 | 8 | 4.02 | 12.8 | 0.7 | 74 | 0 | 9.9 | 5.2 | 80 | 95 | 31 | 49 | 0.01 |
| Pig-#2 | 137.3 | 5.1 | 102.6 | 8.6 | 4.5 | 12.9 | 1 | 99 | 0 | 11.1 | 5.5 | 103 | 88 | 32 | 34 | 0 |
| Pig-#3 | 135.7 | 5.3 | 100.9 | 8.4 | 4 | 12.2 | 1 | 87 | 0 | 10.3 | 4.3 | 69 | 49 | 21 | 29 | 0.01 |
| Pig-#4 | 137.4 | 4.1 | 99.8 | 6.6 | 3.87 | 11.7 | 1 | 84 | 0 | 10.4 | 6.2 | 78 | 22 | 27 | 42 | 0 |

* Na: Sodium, K: Potassium, CL: Chloride, TP: Total Protein, ALB: Albumin, BUN: Blood Urea Nitrogen, CRE: Creatinine, GLU: Glucose TBIL: Total Bilirubin, Ca: Calcium, PHOS: phosphorous, TCHOL: Total Cholesterol, TG: Triglyceride, AST: Aspartate aminotransferase, ALT: Alanine aminotransferase, CRP: C-reactive protein

**Supplementary Table 3**. The results for complete blood cell count in 2H 2019

|  | WBC  (×10^3 cells/µL) | RBC  (×10^6 cells/µL) | HGB  (g/dL) | HCT  (%) | MCV  (fL) | MCH  (pg) | MCHC  (g/dL) | RDW  (%) | HDW  (g/dL) | PLT  (×10^3 cells/uL) | MPV  (fL) | NEUT  (%) | LYM  (%) | MONO  (%) | EOS  (%) | LUC  (%) | BASO  (%) | NEUT  (×10^3 cells/µL) | LYM  (×10^3 cells/µL) | MONO  (×10^3 cells/µL) | EOS  (×10^3 cells/µL) | LUC  (×10^3 cells/µL) | BASO  (×10^3 cells/µL) | Retic  (×10^9 cells/L) | Retic  (%) |
| --- | --- | --- | --- | --- | --- | --- | --- | --- | --- | --- | --- | --- | --- | --- | --- | --- | --- | --- | --- | --- | --- | --- | --- | --- | --- |
| Dog-#1 | 14.34 | 6.09 | 13.1 | 42 | 69 | 21.6 | 31.3 | 15.1 | 2.11 | 502 | 11.9 | 64.5 | 15.6 | 18.7 | 0.2 | 0.9 | 0.1 | 9.24 | 2.24 | 2.68 | 0.03 | 0.13 | 0.01 | 210.7 | 3.46 |
| Dog-#2 | 8.39 | 8.33 | 18 | 57 | 68.4 | 21.5 | 31.5 | 11.9 | 1.62 | 226 | 10.5 | 59.5 | 30.2 | 6.3 | 2.9 | 0.6 | 0.4 | 4.99 | 2.54 | 0.53 | 0.25 | 0.05 | 0.03 | 51.5 | 0.62 |
| Dog-#10 | 9.33 | 7.5 | 15.9 | 49.4 | 65.9 | 21.2 | 32.1 | 13.3 | 1.85 | 496 | 10.1 | 71.1 | 19.4 | 5.7 | 3 | 0.6 | 0.2 | 6.63 | 1.81 | 0.53 | 0.28 | 0.06 | 0.02 | 78.5 | 1.05 |
| Dog-#15 | 11.54 | 7.75 | 15.9 | 51.3 | 66.2 | 20.5 | 31 | 12.1 | 1.73 | 347 | 14.5 | 5.4 | 3.8 | 90.7 | 0 | 0 | 0.2 | 0.62 | 0.44 | 10.46 | 0 | 0 | 0.02 | 70.2 | 0.91 |
| Dog-#16 | 16.27 | 5.66 | 13.2 | 41.3 | 73 | 23.3 | 32 | 17.1 | 2.17 | 382 | 13.9 | 79.1 | 10.7 | 8.4 | 0.3 | 1.3 | 0.1 | 12.87 | 1.74 | 1.37 | 0.05 | 0.21 | 0.02 | 296.9 | 5.25 |
| Dog-#17 | 7.9 | 8.97 | 18.8 | 59.7 | 66.5 | 20.9 | 31.5 | 12.8 | 1.84 | 314 | 14.3 | 56.3 | 35 | 6.6 | 1.6 | 0.5 | 0.2 | 4.45 | 2.76 | 0.52 | 0.12 | 0.04 | 0.01 | 78.2 | 0.87 |
| Dog-#18 | 10.55 | 7.84 | 17.1 | 52.9 | 67.5 | 21.8 | 32.3 | 12.3 | 2.16 | 289 | 11.6 | 60.1 | 29.6 | 5.4 | 4 | 0.6 | 0.3 | 6.34 | 3.12 | 0.57 | 0.42 | 0.07 | 0.03 | 55.2 | 0.7 |
| Dog-#19 | 10.88 | 7.24 | 15.1 | 47.3 | 65.4 | 20.8 | 31.9 | 12.8 | 1.86 | 324 | 10.8 | 67.9 | 17.4 | 6.2 | 7.8 | 0.4 | 0.2 | 7.39 | 1.9 | 0.67 | 0.85 | 0.05 | 0.02 | 46.2 | 0.64 |
| Dog-#21 | 14.45 | 7.53 | 16.8 | 53.5 | 71 | 22.3 | 31.4 | 11.5 | 1.65 | 368 | 15.2 | 75.7 | 14.4 | 8.7 | 0.3 | 0.8 | 0.1 | 10.93 | 2.08 | 1.26 | 0.05 | 0.12 | 0.01 | 26.1 | 0.35 |
| Dog-#22 | 15.91 | 6.77 | 14.2 | 46.6 | 68.8 | 21 | 30.6 | 12.3 | 1.65 | 344 | 15.6 | 76.2 | 13.6 | 6.5 | 3.2 | 0.4 | 0.1 | 12.13 | 2.16 | 1.03 | 0.51 | 0.07 | 0.02 | 53.9 | 0.8 |
| Dog-#23 | 8.78 | 7.14 | 15.8 | 50.5 | 70.7 | 22.1 | 31.3 | 12.1 | 1.73 | 438 | 13 | 60.3 | 31.5 | 6.1 | 1.3 | 0.6 | 0.3 | 5.29 | 2.76 | 0.54 | 0.11 | 0.05 | 0.03 | 69.5 | 0.97 |
| Dog-#24 | 13.49 | 7.37 | 16 | 50.9 | 69.1 | 21.7 | 31.5 | 12.1 | 1.74 | 343 | 14.2 | 63.6 | 22.1 | 7.1 | 6.4 | 0.4 | 0.3 | 8.58 | 2.98 | 0.96 | 0.87 | 0.06 | 0.03 | 85.1 | 1.16 |
| Dog-#25 | 9.52 | 8.14 | 17.1 | 56 | 68.8 | 21 | 30.6 | 12.4 | 1.77 | 351 | 14.1 | 66.9 | 24.4 | 4.8 | 3.5 | 0.3 | 0.2 | 6.36 | 2.32 | 0.45 | 0.33 | 0.03 | 0.02 | 128.9 | 1.58 |
| Dog-#26 | 10.39 | 7.51 | 16.6 | 51.7 | 68.8 | 22.1 | 32.2 | 13.1 | 1.93 | 373 | 9.5 | 62.8 | 28.3 | 4.6 | 3.6 | 0.5 | 0.2 | 6.52 | 2.94 | 0.48 | 0.37 | 0.06 | 0.02 | 67.1 | 0.89 |
| Dog-#28 | 8.41 | 7.86 | 17.4 | 54.5 | 69.3 | 22.2 | 32 | 12 | 1.65 | 268 | 9.7 | 48.2 | 41.1 | 5.9 | 3.7 | 0.7 | 0.4 | 4.05 | 3.46 | 0.5 | 0.31 | 0.06 | 0.03 | 97.6 | 1.24 |
| Dog-#30 | 10.15 | 7.49 | 16.4 | 51.7 | 69 | 21.9 | 31.7 | 12.3 | 1.77 | 272 | 11.5 | 44 | 46.7 | 4.2 | 4.1 | 0.6 | 0.3 | 4.47 | 4.74 | 0.43 | 0.41 | 0.06 | 0.03 | 41.5 | 0.55 |
| Dog-#32 | 9.77 | 7.31 | 15.6 | 50.5 | 69 | 21.3 | 30.9 | 12 | 1.68 | 389 | 15.5 | 59.1 | 31.5 | 7 | 2.1 | 0.3 | 0.1 | 5.77 | 3.07 | 0.68 | 0.2 | 0.03 | 0.01 | 61.7 | 0.84 |
| Dog-#33 | 11.43 | 8.84 | 17.4 | 55.7 | 63 | 19.7 | 31.2 | 12 | 1.88 | 556 | 12 | 49.3 | 23.2 | 20.6 | 6 | 0.4 | 0.4 | 5.64 | 2.65 | 2.35 | 0.69 | 0.05 | 0.05 | 67.7 | 0.77 |
| Dog-#36 | 9.68 | 8.02 | 17.2 | 55 | 68.6 | 21.5 | 31.3 | 12.6 | 1.79 | 305 | 16.9 | 58.1 | 33.3 | 5.5 | 2.3 | 0.5 | 0.2 | 5.63 | 3.23 | 0.53 | 0.22 | 0.05 | 0.02 | 69.4 | 0.87 |
| Dog-#37 | 7.41 | 7.83 | 17.4 | 53.8 | 68.7 | 22.2 | 32.3 | 12.4 | 1.72 | 342 | 13.6 | 57.6 | 34 | 6.2 | 1.4 | 0.6 | 0.2 | 4.27 | 2.52 | 0.46 | 0.1 | 0.05 | 0.01 | 102.7 | 1.31 |
| Dog-#38 | 12.23 | 7.35 | 16.4 | 52.6 | 71.5 | 22.3 | 31.2 | 13.3 | 1.77 | 417 | 15.4 | 5.6 | 4 | 90.1 | 0 | 0 | 0.2 | 0.69 | 0.49 | 11.02 | 0 | 0 | 0.03 | 133.9 | 1.82 |
| Dog-#43 | 8.96 | 7.77 | 17.5 | 54.2 | 69.7 | 22.6 | 32.4 | 12.4 | 1.67 | 254 | 11.1 | 50.8 | 36.4 | 5.2 | 6.7 | 0.6 | 0.4 | 4.55 | 3.26 | 0.47 | 0.6 | 0.05 | 0.03 | 63.6 | 0.82 |
| Dog-#46 | 10.11 | 8.46 | 18.6 | 57.1 | 67.5 | 22 | 32.6 | 12.2 | 1.96 | 217 | 12.9 | 64 | 26.9 | 6.6 | 1.8 | 0.4 | 0.3 | 6.47 | 2.72 | 0.66 | 0.18 | 0.04 | 0.03 | 73 | 0.86 |
| Dog-#47 | 12.94 | 7.83 | 17.4 | 55.2 | 70.5 | 22.2 | 31.6 | 11.6 | 1.56 | 368 | 9.6 | 58.4 | 30.3 | 6.2 | 4 | 0.7 | 0.4 | 7.56 | 3.91 | 0.8 | 0.51 | 0.09 | 0.06 | 47 | 0.6 |
| Pig-#5 | 14.43 | 9.46 | 15.4 | 50.8 | 53.7 | 16.3 | 30.4 | 15.3 | 1.95 | 270 | 10.6 | 19 | 74.6 | 2.1 | 2.2 | 1.6 | 0.5 | 2.74 | 10.76 | 0.31 | 0.32 | 0.23 | 0.07 | 196.1 | 2.07 |
| Pig-#6 | 14.35 | 8.92 | 16.9 | 54.7 | 61.3 | 19 | 30.9 | 13.9 | 1.69 | 232 | 10.7 | 25.8 | 64.2 | 3.1 | 4.4 | 2.1 | 0.4 | 3.7 | 9.21 | 0.45 | 0.63 | 0.3 | 0.06 | 66.6 | 0.75 |
| Pig-#7 | 15.32 | 8 | 15.6 | 50.1 | 62.7 | 19.6 | 31.2 | 14.2 | 1.7 | 402 | 9.2 | 37.7 | 52.3 | 4 | 3.3 | 2.4 | 0.2 | 5.78 | 8.01 | 0.62 | 0.51 | 0.37 | 0.03 | 59.6 | 0.75 |
| Pig-#8 | 13.66 | 9.73 | 18.9 | 61.2 | 62.9 | 19.5 | 31 | 15.2 | 1.78 | 232 | 9.5 | 28.4 | 53.1 | 4 | 12.2 | 1.8 | 0.5 | 3.88 | 7.26 | 0.54 | 1.67 | 0.24 | 0.06 | 157.4 | 1.62 |
| Pig-#9 | 12.57 | 8.18 | 15.6 | 51.5 | 63 | 19 | 30.2 | 15.8 | 1.9 | 277 | 10.3 | 29.3 | 55.6 | 4.6 | 6.9 | 3.3 | 0.3 | 3.68 | 6.99 | 0.57 | 0.87 | 0.42 | 0.03 | 108.5 | 1.33 |
| Pig-#10 | 13.37 | 9.35 | 15.9 | 54.8 | 58.6 | 17 | 29 | 16.1 | 1.85 | 16 | 21.8 | 25.8 | 60 | 3.8 | 7.9 | 1.7 | 0.8 | 3.44 | 8.03 | 0.51 | 1.06 | 0.22 | 0.11 | 205 | 2.19 |
| Pig-#11 | 8.41 | 7.72 | 15.4 | 49.5 | 64.1 | 20 | 31.2 | 14.8 | 1.83 | 280 | 9.1 | 36.6 | 54.7 | 4 | 2.6 | 1.8 | 0.3 | 3.08 | 4.6 | 0.33 | 0.22 | 0.15 | 0.02 | 80.1 | 1.04 |

* WBC: White blood cell, RBC: Red blood cell, HGB: Hemoglobin, HCT: hematocrit, MCV: mean corpuscular volume, MCH: mean corpuscular hemoglobin, MCHC: mean corpuscular hemoglobin concentration, RDW: Red Blood Cell Distribution Width, HDW: hemoglobin distribution width, PLT: platelet, MPV: Mean platelet volume, NEUT: Neutrophil, LYM: Lymphocyte, MONO: Monocyte, EOS: Eosinophil, LUC: Large unstained cell, Retic: Reticulocyte.

**Supplementary Table 4**. The results for blood biochemistry in 2H 2019

| Sample Name | Na (mmol/L) | K (mmol/L) | CL (mmol/L) | TP (g/dL) | ALB (g/dL) | BUN  (mg/dL) | CRE  (mg/dL) | GLU  (mg/dL) | TBIL  (mg/dL) | Ca  (mg/dL) | PHOS  (mg/dL) | TCHOL (mg/dL) | TG (mg/dL) | AST  (U/L) | ALT (U/L) | CRP (mg/dL) |
| --- | --- | --- | --- | --- | --- | --- | --- | --- | --- | --- | --- | --- | --- | --- | --- | --- |
| Dog-#1 | 143.6 | 4.5 | 104.4 | 17 | 4.01 | 20.1 | 0.8 | 52 | 0 | 11 | 3.6 | 198 | 61 | 58 | 53 | 0 |
| Dog-#2 | 146.7 | 4.6 | 110.4 | 8.5 | 3.23 | 21.7 | 0.7 | 73 | 0 | 10.4 | 4 | 244 | 109 | 32 | 28 | 0.004 |
| Dog-#10 | 148.1 | 4.6 | 110.4 | 7.4 | 3.12 | 30 | 0.8 | 69 | 0 | 10 | 3.6 | 141 | 79 | 43 | 46 | 0 |
| Dog-#15 | 145.2 | 4.5 | 100 | 11.7 | 3.89 | 27.6 | 0.5 | 34 | 0 | 10.2 | 5.2 | 115 | 226 | 46 | 55 | 0 |
| Dog-#16 | 146.5 | 4.4 | 98.8 | 8.7 | 3.48 | 16.4 | 0.5 | 43 | 0 | 10.1 | 3.4 | 141 | 139 | 43 | 48 | 0.001 |
| Dog-#17 | 147.9 | 4.5 | 98.3 | 10.2 | 3.86 | 17.5 | 0.5 | 73 | 0 | 10.9 | 5 | 162 | 189 | 53 | 287 | 0 |
| Dog-#18 | 144.6 | 4.8 | 107.9 | 7.6 | 3.48 | 26.3 | 0.8 | 50 | 0 | 10.8 | 4.5 | 149 | 57 | 37 | 49 | 0 |
| Dog-#19 | 149.2 | 3.9 | 109.2 | 7.1 | 3.15 | 27.8 | 0.8 | 79 | 0 | 10.7 | 4.3 | 163 | 65 | 31 | 46 | 0.001 |
| Dog-#21 | 147.3 | 4.4 | 110.1 | 7.5 | 3.25 | 25.3 | 0.8 | 79 | 0 | 10.8 | 3.9 | 164 | 73 | 40 | 50 | 0.002 |
| Dog-#22 | 147 | 4.7 | 106.2 | 8.9 | 3.32 | 24 | 0.9 | 54 | 0 | 10.6 | 4 | 154 | 91 | 56 | 37 | 0.001 |
| Dog-#23 | 145.8 | 4.3 | 96.7 | 8.1 | 3.44 | 16 | 0.6 | 56 | 0 | 10.4 | 3.6 | 89 | 91 | 50 | 105 | 0.01 |
| Dog-#24 | 145.4 | 4.3 | 107.4 | 7.6 | 2.84 | 16.8 | 0.6 | 53 | 0 | 10.2 | 3.8 | 174 | 55 | 31 | 33 | 0.042 |
| Dog-#25 | 145.9 | 4.6 | 103.8 | 8.4 | 3.39 | 19.7 | 0.9 | 63 | 0 | 10.5 | 4.4 | 189 | 68 | 42 | 45 | 0.004 |
| Dog-#26 | 145.9 | 5 | 108.8 | 6.9 | 3.09 | 27.9 | 1 | 82 | 0 | 10.1 | 3 | 162 | 35 | 55 | 60 | 0.009 |
| Dog-#28 | 148.8 | 4.2 | 106.2 | 7.5 | 3.46 | 32.8 | 0.9 | 62 | 0 | 11.2 | 4.1 | 173 | 65 | 39 | 38 | 0 |
| Dog-#30 | 147.3 | 4.3 | 110.1 | 7.5 | 3.39 | 17.8 | 0.9 | 91 | 0.1 | 10.8 | 5.7 | 134 | 29 | 37 | 55 | 0.003 |
| Dog-#32 | 145.5 | 4.6 | 108.3 | 7 | 3.44 | 15.4 | 0.7 | 92 | 0.1 | 11 | 5.5 | 141 | 32 | 41 | 40 | 0 |
| Dog-#33 | 146.3 | 4.7 | 109.2 | 8.3 | 3.17 | 19.5 | 0.8 | 57 | 0 | 10.5 | 3.7 | 195 | 92 | 42 | 67 | 0.003 |
| Dog-#36 | 147.9 | 4.5 | 105.7 | 8.9 | 3.2 | 19.4 | 0.8 | 56 | 0 | 10.2 | 4.5 | 157 | 112 | 51 | 46 | 0 |
| Dog-#37 | 147.2 | 4.9 | 108.8 | 7.7 | 3.27 | 26 | 0.9 | 42 | 0.1 | 10.4 | 3.6 | 137 | 39 | 52 | 45 | 0 |
| Dog-#38 | 146.9 | 4.7 | 108.8 | 10 | 3.47 | 20.8 | 0.7 | 46 | 0 | 10.9 | 3.3 | 202 | 132 | 44 | 31 | 0 |
| Dog-#43 | 149.6 | 4.3 | 102.2 | 10.8 | 3.35 | 15 | 0.6 | 33 | 0 | 11.1 | 4.6 | 179 | 121 | 43 | 40 | 0 |
| Dog-#46 | 149.7 | 4.4 | 107 | 6.9 | 2.92 | 14.3 | 0.6 | 37 | 0 | 10.2 | 3.9 | 113 | 28 | 49 | 30 | 0.022 |
| Dog-#47 | 147.2 | 5 | 108.3 | 8.2 | 3.21 | 23 | 0.6 | 69 | 0 | 10.9 | 4.4 | 184 | 84 | 50 | 44 | 0 |
| Pig-#5 | 145.5 | 6 | 105.2 | 7.8 | 3.9 | 11 | 2.6 | 84 | 0 | 11.3 | 7.3 | 103 | 58 | 38 | 32 | 0.018 |
| Pig-#6 | 145.7 | 5 | 105.8 | 7.4 | 4.51 | 8.3 | 1 | 70 | 0 | 10.3 | 7 | 55 | 19 | 33 | 41 | 0.008 |
| Pig-#7 | 143.2 | 6.2 | 101.7 | 8.5 | 4.49 | 11.3 | 1.1 | 72 | 0 | 10.9 | 8.1 | 98 | 45 | 23 | 45 | 0.023 |
| Pig-#8 | 153.6 | 6.6 | 108.9 | 10.7 | 4.83 | 13.7 | 1 | 88 | 0 | 11.4 | 8.3 | 99 | 37 | 181 | 48 | 0.005 |
| Pig-#9 | 139.5 | 5.3 | 104.9 | 7.4 | 4.48 | 8.9 | 0.9 | 75 | 0 | 10 | 7.7 | 63 | 13 | 259 | 38 | 0.004 |
| Pig-#10 | 150.2 | 6.4 | 107.7 | 8.1 | 4.5 | 11.4 | 1.4 | 171 | 0 | 10.9 | 9.4 | 80 | 26 | 55 | 31 | 0.02 |
| Pig-#11 | 139.8 | 4.4 | 103.3 | 7.6 | 4.21 | 10.6 | 1 | 91 | 0 | 9.8 | 6.8 | 81 | 29 | 68 | 38 | 0 |
| Dog-#36 | 142.3 | 4.6 | 104.5 | 6.2 | 2.96 | 28.2 | 0.7 | 77 | 0 | 11.1 | 4.4 | 170 | 86 | 34 | 31 | 0.01 |
| Dog-#24 | 137.8 | 4.3 | 100 | 6.5 | 3.32 | 19.6 | 0.8 | 86 | 0.2 | 11.1 | 6.2 | 136 | 24 | 35 | 28 | 0.01 |
| Dog-#26 | 134.1 | 4.3 | 102.5 | 6.5 | 3.14 | 20.4 | 0.7 | 100 | 0.2 | 10.8 | 5.6 | 167 | 27 | 31 | 22 | 0.02 |
| Dog-#9 | 140.8 | 4.6 | 109.2 | 5.1 | 2.84 | 16.6 | 0.6 | 91 | 0 | 10.3 | 6.7 | 129 | 24 | 27 | 26 | 0 |
| Dog-#37 | 142.5 | 5 | 102.9 | 5.8 | 3.07 | 26.7 | 0.8 | 72 | 0 | 11.2 | 5.8 | 136 | 70 | 34 | 36 | 0.01 |
| Dog-#34 | 140.4 | 4.6 | 102.4 | 6.9 | 3.18 | 21.4 | 0.7 | 78 | 0.1 | 11.3 | 5.2 | 171 | 97 | 48 | 27 | 0 |
| Dog-#22 | 145.2 | 4.2 | 101.7 | 6.3 | 3.28 | 18.4 | 0.8 | 62 | 0.1 | 11.5 | 7.6 | 194 | 27 | 51 | 51 | 0 |
| Dog-#5 | 140.4 | 4.7 | 106 | 6.2 | 3.14 | 20.5 | 0.8 | 89 | 0.1 | 10.5 | 6 | 138 | 24 | 31 | 32 | 0 |
| Dog-#3 | 143.3 | 4.6 | 103 | 6.3 | 3.1 | 30.5 | 0.8 | 89 | 0 | 12.6 | 4.5 | 173 | 121 | 31 | 24 | 0 |
| Dog-#4 | 140.9 | 4.6 | 104.1 | 6 | 3.1 | 30.8 | 0.8 | 103 | 0 | 12 | 4.6 | 179 | 51 | 30 | 36 | 0.01 |
| Dog-#2 | 144 | 5 | 108.9 | 5.2 | 2.77 | 28.9 | 0.7 | 96 | 0 | 10.9 | 5.3 | 78 | 50 | 36 | 41 | 0 |
| Dog-#35 | 144.5 | 4.4 | 106 | 5.7 | 3.06 | 20.2 | 0.7 | 70 | 0 | 11.3 | 5.6 | 125 | 24 | 37 | 34 | 0.01 |
| Dog-#13 | 142.2 | 4.6 | 107.1 | 5.8 | 3 | 11.9 | 0.6 | 87 | 0 | 11.1 | 6.3 | 160 | 49 | 29 | 17 | 0 |
| Dog-#33 | 144.4 | 5 | 102.2 | 6.1 | 3.09 | 21.7 | 0.7 | 72 | 0 | 11.2 | 6 | 184 | 76 | 48 | 37 | 0 |
| Pig-#1 | 135.8 | 4.2 | 99.1 | 8 | 4.02 | 12.8 | 0.7 | 74 | 0 | 9.9 | 5.2 | 80 | 95 | 31 | 49 | 0.01 |
| Pig-#2 | 137.3 | 5.1 | 102.6 | 8.6 | 4.5 | 12.9 | 1 | 99 | 0 | 11.1 | 5.5 | 103 | 88 | 32 | 34 | 0 |
| Pig-#3 | 135.7 | 5.3 | 100.9 | 8.4 | 4 | 12.2 | 1 | 87 | 0 | 10.3 | 4.3 | 69 | 49 | 21 | 29 | 0.01 |
| Pig-#4 | 137.4 | 4.1 | 99.8 | 6.6 | 3.87 | 11.7 | 1 | 84 | 0 | 10.4 | 6.2 | 78 | 22 | 27 | 42 | 0 |

* Na: Sodium, K: Potassium, CL: Chloride, TP: Total Protein, ALB: Albumin, BUN: Blood Urea Nitrogen, CRE: Creatinine, GLU: Glucose TBIL: Total Bilirubin, Ca: Calcium, PHOS: phosphorous, TCHOL: Total Cholesterol, TG: Triglyceride, AST: Aspartate aminotransferase, ALT: Alanine aminotransferase, CRP: C-reactive protein

**Supplementary Table 5**. The results for complete blood cell count in 1H 2020

| Sample Name | WBC  (×10^3 cells/µL) | RBC  (×10^6 cells/µL) | HGB  (g/dL) | HCT  (%) | MCV  (fL) | MCH  (pg) | MCHC  (g/dL) | RDW  (%) | HDW  (g/dL) | PLT  (×10^3 cells/uL) | MPV  (fL) | NEUT  (%) | LYM  (%) | MONO  (%) | EOS  (%) | LUC  (%) | BASO  (%) | NEUT  (×10^3 cells/µL) | LYM  (×10^3 cells/µL) | MONO  (×10^3 cells/µL) | EOS  (×10^3 cells/µL) | LUC  (×10^3 cells/µL) | BASO  (×10^3 cells/µL) | Retic  (×10^9 cells/L) | Retic  (%) |
| --- | --- | --- | --- | --- | --- | --- | --- | --- | --- | --- | --- | --- | --- | --- | --- | --- | --- | --- | --- | --- | --- | --- | --- | --- | --- |
| Dog-#1 | 10.76 | 7.46 | 17 | 50.2 | 67.3 | 22.8 | 33.9 | 12.3 | 1.74 | 241 | 9.7 | 74 | 17.6 | 6.3 | 1.6 | 0.4 | 0.1 | 7.96 | 1.9 | 0.68 | 0.17 | 0.05 | 0.01 | 74.4 | 1 |
| Dog-#2 | 5.35 | 8.96 | 19.2 | 57.4 | 64.1 | 21.4 | 33.4 | 11.7 | 1.73 | 297 | 10 | 61.5 | 29.1 | 3.7 | 4.3 | 0.9 | 0.6 | 3.29 | 1.56 | 0.2 | 0.23 | 0.05 | 0.03 | 24.3 | 0.27 |
| Dog-#10 | 7.8 | 7.88 | 17.7 | 53.1 | 67.4 | 22.5 | 33.3 | 11.9 | 1.73 | 335 | 8.7 | 64.4 | 28.2 | 3.1 | 3.8 | 0.5 | 0.1 | 5.02 | 2.2 | 0.24 | 0.29 | 0.04 | 0.01 | 59.4 | 0.75 |
| Dog-#14 | 10.14 | 5.95 | 13.4 | 40.8 | 68.6 | 22.5 | 32.7 | 13.4 | 1.72 | 338 | 11.8 | 64.3 | 26.9 | 6.2 | 1.8 | 0.6 | 0.2 | 6.52 | 2.73 | 0.63 | 0.18 | 0.06 | 0.02 | 87.3 | 1.47 |
| Dog-#15 | 10.14 | 7.17 | 15 | 45.4 | 63.3 | 20.9 | 33.1 | 17 | 2.12 | 319 | 10.4 | 60.2 | 32.9 | 4.7 | 1.5 | 0.5 | 0.1 | 6.11 | 3.34 | 0.48 | 0.15 | 0.05 | 0.01 | 69.4 | 0.97 |
| Dog-#16 | 7.49 | 7.53 | 16.2 | 48.8 | 64.8 | 21.5 | 33.1 | 12.8 | 1.93 | 241 | 10 | 61.4 | 30.2 | 5.7 | 1.6 | 0.8 | 0.3 | 4.6 | 2.26 | 0.43 | 0.12 | 0.06 | 0.02 | 52.1 | 0.69 |
| Dog-#17 | 11.82 | 6.53 | 14.2 | 44.5 | 68.2 | 21.7 | 31.9 | 13.7 | 1.73 | 342 | 10.6 | 52.5 | 36.2 | 8.4 | 2 | 0.9 | 0.2 | 6.2 | 4.28 | 0.99 | 0.23 | 0.11 | 0.02 | 101.8 | 1.56 |
| Dog-#18 | 8.78 | 6.98 | 15.3 | 43.4 | 62.1 | 22 | 35.4 | 13.8 | 3.03 | 279 | 10.4 | 62.8 | 28.6 | 3.7 | 3.9 | 0.6 | 0.3 | 5.51 | 2.51 | 0.33 | 0.34 | 0.06 | 0.03 | 70.4 | 1.01 |
| Dog-#19 | 10.34 | 6.86 | 15.8 | 44.8 | 65.2 | 23 | 35.2 | 13.1 | 2.95 | 245 | 10.6 | 51.3 | 40.9 | 4.2 | 2.1 | 1 | 0.5 | 5.31 | 4.22 | 0.43 | 0.21 | 0.11 | 0.05 | 54.4 | 0.79 |
| Dog-#20 | 6.7 | 7.55 | 17.4 | 48.4 | 64.1 | 23.1 | 36 | 13 | 3.56 | 253 | 8.9 | 50.4 | 37.3 | 6.3 | 4.2 | 1.2 | 0.6 | 3.38 | 2.5 | 0.42 | 0.28 | 0.08 | 0.04 | 97.4 | 1.29 |
| Dog-#21 | 8.45 | 6.88 | 16 | 47.2 | 68.6 | 23.3 | 34 | 12.3 | 1.9 | 282 | 11.1 | 53 | 31.2 | 6.2 | 8.5 | 0.9 | 0.2 | 4.48 | 2.64 | 0.52 | 0.72 | 0.07 | 0.02 | 77.4 | 1.12 |
| Dog-#22 | 7.82 | 7.38 | 16.3 | 49.3 | 66.8 | 22.1 | 33.1 | 12.5 | 1.78 | 305 | 10.2 | 67.7 | 24 | 4.4 | 3.5 | 0.4 | 0.1 | 5.29 | 1.88 | 0.34 | 0.27 | 0.03 | 0.01 | 103.9 | 1.41 |
| Dog-#24 | 8.37 | 7.75 | 17.5 | 53.3 | 68.7 | 22.6 | 32.8 | 13.3 | 1.9 | 300 | 11.3 | 66 | 25.5 | 5 | 2.7 | 0.6 | 0.2 | 5.52 | 2.13 | 0.42 | 0.22 | 0.05 | 0.02 | 80.4 | 1.04 |
| Dog-#25 | 9.35 | 7.04 | 16.2 | 48.4 | 68.7 | 23 | 33.5 | 14.4 | 2.02 | 370 | 10.4 | 62 | 26.5 | 4.2 | 6.5 | 0.7 | 0.3 | 5.79 | 2.48 | 0.39 | 0.6 | 0.06 | 0.02 | 128.3 | 1.82 |
| Dog-#28 | 9.74 | 7.5 | 17 | 48.1 | 64.1 | 22.7 | 35.5 | 13.4 | 3.79 | 278 | 10 | 67.4 | 22.5 | 4.6 | 4.1 | 0.7 | 0.7 | 6.57 | 2.19 | 0.45 | 0.4 | 0.07 | 0.07 | 103.1 | 1.37 |
| Dog-#30 | 7.25 | 8.02 | 17.8 | 50 | 62.3 | 22.2 | 35.7 | 12.8 | 4.12 | 184 | 10.8 | 60.2 | 29.2 | 6.1 | 3 | 0.9 | 0.6 | 4.36 | 2.12 | 0.44 | 0.22 | 0.07 | 0.04 | 77 | 0.96 |
| Dog-#32 | 10.64 | 7.34 | 16.8 | 50 | 68.2 | 22.9 | 33.6 | 12.4 | 1.99 | 307 | 10.2 | 54.7 | 30.8 | 5 | 8.6 | 0.6 | 0.2 | 5.82 | 3.28 | 0.54 | 0.92 | 0.07 | 0.02 | 68 | 0.93 |
| Dog-#36 | 13.6 | 6.86 | 15 | 44.9 | 65.5 | 21.8 | 33.4 | 12.2 | 1.77 | 295 | 9.6 | 69.8 | 21.7 | 4.8 | 3.1 | 0.5 | 0.1 | 9.5 | 2.95 | 0.65 | 0.42 | 0.07 | 0.02 | 78.7 | 1.15 |
| Dog-#37 | 11.57 | 6.9 | 15.4 | 42.5 | 61.6 | 22.3 | 36.1 | 16.4 | 3.82 | 90 | 9.3 | 63.5 | 26.1 | 5.3 | 3.9 | 0.8 | 0.3 | 7.35 | 3.02 | 0.62 | 0.45 | 0.09 | 0.03 | 111.6 | 1.62 |
| Dog-#46 | 11.25 | 7.35 | 16.4 | 49.2 | 67 | 22.3 | 33.4 | 12.5 | 1.89 | 172 | 11.5 | 68.8 | 22.6 | 5.1 | 2.7 | 0.6 | 0.2 | 7.74 | 2.54 | 0.58 | 0.3 | 0.07 | 0.02 | 58.4 | 0.79 |
| Dog-#47 | 11.07 | 7.09 | 15.6 | 43.5 | 61.3 | 21.9 | 35.8 | 15.8 | 3.1 | 298 | 9.7 | 60.2 | 29.1 | 7 | 2.3 | 1.1 | 0.4 | 6.66 | 3.22 | 0.78 | 0.25 | 0.13 | 0.04 | 80.3 | 1.13 |
| Dog-#48 | 10.21 | 7.9 | 18.3 | 53.5 | 67.7 | 23.2 | 34.3 | 12.6 | 1.96 | 250 | 10.8 | 62.3 | 28.3 | 5.1 | 3.2 | 0.6 | 0.4 | 6.36 | 2.89 | 0.52 | 0.33 | 0.06 | 0.05 | 88.8 | 1.12 |
| Pig-#10 | 8.88 | 7.68 | 15.8 | 48.1 | 62.7 | 20.6 | 32.9 | 15.4 | 1.85 | 191 | 9.1 | 30.5 | 62 | 2.5 | 2.2 | 2.6 | 0.2 | 2.71 | 5.51 | 0.22 | 0.2 | 0.23 | 0.02 | 88.6 | 1.15 |
| Pig-#11 | 14.29 | 8.74 | 15.2 | 46.1 | 52.8 | 17.4 | 32.9 | 15.8 | 2.14 | 233 | 9.7 | 35.7 | 48.3 | 3.2 | 9.5 | 3.2 | 0.1 | 5.1 | 6.9 | 0.46 | 1.36 | 0.46 | 0.01 | 77.5 | 0.89 |
| Pig-#12 | 19.59 | 8.83 | 14.7 | 47 | 53.2 | 16.6 | 31.3 | 14.8 | 1.76 | 274 | 9.8 | 28.1 | 61.8 | 3.3 | 4.2 | 2.6 | 0.1 | 5.5 | 12.09 | 0.64 | 0.82 | 0.5 | 0.03 | 119.4 | 1.35 |
| Pig-#13 | 14.42 | 7.97 | 16.4 | 51.5 | 64.6 | 20.6 | 31.9 | 13.6 | 1.66 | 339 | 8.6 | 21.3 | 65.6 | 4.8 | 4.4 | 3.7 | 0.2 | 3.08 | 9.46 | 0.69 | 0.64 | 0.53 | 0.03 | 85.8 | 1.08 |
| Pig-#14 | 11.7 | 7.59 | 16.6 | 49.3 | 65 | 21.8 | 33.5 | 13.7 | 1.64 | 249 | 10.1 | 25.7 | 61.3 | 2.8 | 7.6 | 2.4 | 0.2 | 3.01 | 7.17 | 0.33 | 0.89 | 0.28 | 0.02 | 108.1 | 1.42 |
| Pig-#15 | 12.74 | 9.15 | 17.6 | 53.1 | 58.1 | 19.2 | 33.1 | 14.7 | 2.03 | 250 | 8.4 | 26.6 | 64.8 | 3.5 | 2 | 3 | 0.2 | 3.39 | 8.25 | 0.44 | 0.26 | 0.38 | 0.02 | 102.4 | 1.12 |
| Pig-#16 | 10.58 | 7.7 | 16 | 50.2 | 65.2 | 20.8 | 31.8 | 14.5 | 1.68 | 286 | 8.8 | 29.7 | 61.4 | 2.5 | 3.7 | 2.6 | 0.1 | 3.14 | 6.5 | 0.27 | 0.39 | 0.28 | 0.01 | 78.3 | 1.02 |
| Pig-#17 | 12.3 | 6.79 | 15.6 | 46.1 | 68 | 23 | 33.9 | 14 | 1.65 | 298 | 8.8 | 31.4 | 56.3 | 3.7 | 4.1 | 4.2 | 0.3 | 3.87 | 6.93 | 0.46 | 0.5 | 0.52 | 0.03 | 31.7 | 0.47 |
| Pig-#18 | 13.9 | 8.19 | 16.2 | 49.9 | 61 | 19.7 | 32.4 | 14.9 | 1.89 | 211 | 10.2 | 35.9 | 54.1 | 2.1 | 5.3 | 2.5 | 0.1 | 5 | 7.53 | 0.29 | 0.73 | 0.34 | 0.02 | 104.6 | 1.28 |
| Pig-#19 | 9.59 | 7.79 | 16.6 | 50.9 | 65.4 | 21.3 | 32.5 | 13.9 | 1.71 | 207 | 10.1 | 30.3 | 56.1 | 4.5 | 4.5 | 4.3 | 0.3 | 2.9 | 5.38 | 0.43 | 0.43 | 0.41 | 0.03 | 140.4 | 1.8 |
| Pig-#20 | 9.46 | 7.57 | 16.3 | 50.4 | 66.6 | 21.6 | 32.4 | 13.3 | 1.61 | 235 | 9.6 | 19.2 | 69.1 | 6.1 | 4.2 | 1.3 | 0.1 | 1.81 | 6.54 | 0.58 | 0.39 | 0.13 | 0.01 | 74.8 | 0.99 |
| Pig-#21 | 14.72 | 7.18 | 12.9 | 38.8 | 54.1 | 18 | 33.3 | 13.9 | 1.8 | 246 | 10 | 25.4 | 66.3 | 2.9 | 3.2 | 2.1 | 0.1 | 3.74 | 9.75 | 0.43 | 0.47 | 0.31 | 0.02 | 150.9 | 2.1 |
| Pig-#22 | 7.26 | 7.25 | 14.6 | 44.7 | 61.7 | 20.1 | 32.6 | 15.3 | 1.79 | 310 | 8.8 | 32.9 | 52.3 | 7.3 | 0.9 | 6.2 | 0.5 | 2.39 | 3.8 | 0.53 | 0.06 | 0.45 | 0.03 | 97.1 | 1.34 |
| Pig-#23 | 9.7 | 7.54 | 15.7 | 47.5 | 63 | 20.8 | 33 | 15.2 | 1.74 | 332 | 7.9 | 47.7 | 42.1 | 6.6 | 1.1 | 2.3 | 0.1 | 4.63 | 4.09 | 0.64 | 0.11 | 0.22 | 0.01 | 138.6 | 1.84 |

* WBC: White blood cell, RBC: Red blood cell, HGB: Hemoglobin, HCT: hematocrit, MCV: mean corpuscular volume, MCH: mean corpuscular hemoglobin, MCHC: mean corpuscular hemoglobin concentration, RDW: Red Blood Cell Distribution Width, HDW: hemoglobin distribution width, PLT: platelet, MPV: Mean platelet volume, NEUT: Neutrophil, LYM: Lymphocyte, MONO: Monocyte, EOS: Eosinophil, LUC: Large unstained cell, Retic: Reticulocyte.

**Supplementary Table 6**. The results for blood biochemistry in 1H 2020

| Sample Name | Na (mmol/L) | K (mmol/L) | CL (mmol/L) | TP (g/dL) | ALB (g/dL) | BUN  (mg/dL) | CRE  (mg/dL) | GLU  (mg/dL) | TBIL  (mg/dL) | Ca  (mg/dL) | PHOS  (mg/dL) | TCHOL (mg/dL) | TG (mg/dL) | AST  (U/L) | ALT (U/L) | CRP (mg/dL) |
| --- | --- | --- | --- | --- | --- | --- | --- | --- | --- | --- | --- | --- | --- | --- | --- | --- |
| Dog-#1 | 145.5 | 4.7 | 112.5 | 5.8 | 3.14 | 21 | 0.9 | 92 | 0 | 8.2 | 3.9 | 129 | 13 | 36 | 49 | 0 |
| Dog-#2 | 145.2 | 4.9 | 107 | 5.8 | 3.19 | 9.7 | 0.6 | 87 | 0 | 10.4 | 7.4 | 127 | 19 | 31 | 36 | 0.029 |
| Dog-#10 | 146.5 | 4.8 | 110.8 | 6.6 | 3.18 | 25.2 | 0.8 | 82 | 0 | 10.3 | 4.8 | 133 | 41 | 40 | 61 | 0.02 |
| Dog-#15 | 148.6 | 5 | 110.8 | 6.9 | 3.39 | 29.4 | 0.9 | 31 | 0.1 | 10.7 | 6.1 | 213 | 51 | 43 | 53 | 0.006 |
| Dog-#16 | 150.8 | 4.9 | 112.8 | 6.2 | 3.24 | 15.5 | 0.9 | 90 | 0 | 9.4 | 4.9 | 139 | 29 | 46 | 116 | 0.017 |
| Dog-#17 | 144.7 | 4.5 | 108.9 | 6.3 | 3.16 | 18.7 | 1 | 96 | 0 | 9 | 2.7 | 173 | 20 | 43 | 48 | 0.006 |
| Dog-#18 | 147.9 | 4.6 | 108.1 | 6.3 | 3.39 | 13.4 | 0.9 | 68 | 0 | 9.4 | 3.6 | 186 | 21 | 28 | 31 | 0.03 |
| Dog-#19 | 145.6 | 4.8 | 110.4 | 7.2 | 3.33 | 16.4 | 0.9 | 83 | 0.1 | 9.6 | 4.6 | 153 | 41 | 49 | 45 | 0.005 |
| Dog-#20 | 143 | 4.4 | 108.6 | 6.4 | 3.3 | 12.8 | 0.9 | 89 | 0.1 | 9.3 | 4.5 | 155 | 12 | 34 | 53 | 0.011 |
| Dog-#21 | 145.6 | 4.6 | 110.5 | 6.1 | 3 | 13 | 0.8 | 91 | 0 | 9.3 | 4.6 | 184 | 9 | 30 | 49 | 0 |
| Dog-#22 | 146 | 4.8 | 109.3 | 6.2 | 3.21 | 16.7 | 0.9 | 84 | 0 | 9.1 | 4.1 | 125 | 11 | 42 | 55 | 0.025 |
| Dog-#24 | 147.5 | 4.9 | 108.3 | 6.5 | 3.38 | 14.9 | 0.8 | 73 | 0.1 | 10.1 | 5.8 | 117 | 26 | 44 | 49 | 0.006 |
| Dog-#25 | 149.2 | 4.9 | 110.7 | 6.1 | 3.19 | 27 | 0.8 | 56 | 0 | 10.3 | 5.5 | 173 | 23 | 26 | 46 | 0 |
| Dog-#28 | 149.6 | 5.2 | 110.6 | 6.5 | 3.48 | 20.5 | 1 | 86 | 0 | 9.9 | 5 | 155 | 18 | 30 | 39 | 0 |
| Dog-#30 | 145.6 | 4.8 | 106.8 | 5.5 | 3.08 | 8.3 | 0.6 | 93 | 0 | 10.3 | 7.3 | 142 | 15 | 31 | 34 | 0.005 |
| Dog-#32 | 145.9 | 4.8 | 106 | 7 | 3.37 | 20.3 | 0.8 | 77 | 0 | 9.5 | 5.8 | 145 | 15 | 45 | 47 | 0.008 |
| Dog-#36 | 147.6 | 4.9 | 115.7 | 5.9 | 3.2 | 21.4 | 0.9 | 87 | 0 | 9.4 | 6 | 143 | 45 | 41 | 86 | 0 |
| Dog-#37 | 149.7 | 5 | 112.7 | 6.2 | 3.47 | 17.4 | 0.8 | 100 | 0 | 8.8 | 5.1 | 144 | 30 | 30 | 32 | 0.038 |
| Dog-#46 | 146.4 | 4.7 | 111.8 | 5.8 | 3.16 | 23.3 | 0.9 | 90 | 0 | 9.4 | 5.5 | 154 | 35 | 36 | 47 | 0 |
| Dog-#47 | 146.9 | 4.5 | 108.4 | 6.1 | 3.35 | 12 | 0.8 | 91 | 0 | 9 | 4.5 | 165 | 64 | 28 | 40 | 0.025 |
| Dog-#48 | 148 | 4.9 | 113.3 | 7 | 3.46 | 18.2 | 0.9 | 86 | 0.2 | 8.9 | 4.7 | 138 | 15 | 56 | 102 | 0.025 |
| Pig-#10 | 151.1 | 6.7 | 107.3 | 8 | 4.31 | 11.7 | 1.1 | 72 | 0 | 10.4 | 7.8 | 81 | 19 | 30 | 32 | 0 |
| Pig-#11 | 145.2 | 7.2 | 100.6 | 7.4 | 4.28 | 9.3 | 0.8 | 96 | 0 | 10.4 | 8.1 | 75 | 58 | 22 | 29 | 0.042 |
| Pig-#12 | 138.1 | 5.7 | 100.6 | 7.8 | 4.31 | 10.9 | 1.3 | 79 | 0.1 | 9.6 | 7.5 | 89 | 28 | 114 | 38 | 0.024 |
| Pig-#13 | 137.8 | 5.4 | 101.5 | 8.9 | 4.28 | 14.6 | 1.3 | 60 | 0.1 | 10.3 | 6.1 | 96 | 31 | 69 | 45 | 0.029 |
| Pig-#14 | 147.7 | 4.5 | 106.9 | 7.3 | 3.9 | 11 | 2.4 | 135 | 0 | 11.3 | 7 | 89 | 46 | 31 | 41 | 0.011 |
| Pig-#15 | 143.2 | 6.4 | 103.1 | 7.8 | 4.25 | 10.4 | 1.2 | 61 | 0 | 10.1 | 7.6 | 75 | 15 | 29 | 30 | 0 |
| Pig-#16 | 142.2 | 4.7 | 101.3 | 7.5 | 4.27 | 13 | 1.2 | 97 | 0 | 10 | 7.2 | 57 | 74 | 73 | 53 | 0.005 |
| Pig-#17 | 144.4 | 5.3 | 101.6 | 7.6 | 4.16 | 9.8 | 1.2 | 95 | 0 | 10.1 | 6.6 | 60 | 31 | 43 | 46 | 0 |
| Pig-#18 | 145.7 | 5.7 | 103.3 | 6.9 | 4.16 | 7.7 | 1.2 | 70 | 0.1 | 9.9 | 7.9 | 67 | 19 | 38 | 33 | 0 |
| Pig-#19 | 137.4 | 3.8 | 100.8 | 7 | 4 | 14.5 | 1.4 | 123 | 0 | 9.5 | 6.5 | 57 | 29 | 30 | 35 | 0 |
| Pig-#20 | 131.1 | 4.5 | 89.8 | 5.8 | 3.74 | 10.6 | 1.9 | 91 | 0 | 10.3 | 6.5 | 73 | 34 | 32 | 42 | 0.061 |
| Pig-#21 | 139.3 | 4.9 | 101.9 | 7.7 | 4.58 | 9.7 | 1.2 | 96 | 0.1 | 10 | 7.5 | 84 | 31 | 39 | 38 | 0 |
| Pig-#22 | 141.1 | 4.2 | 102.3 | 7.9 | 4.21 | 10.2 | 1.3 | 68 | 0.1 | 10.3 | 7.2 | 91 | 36 | 32 | 27 | 0.037 |
| Pig-#23 | 143.1 | 5.5 | 104.6 | 7.8 | 4.4 | 10.6 | 1.1 | 98 | 0 | 10.4 | 6.3 | 70 | 129 | 61 | 35 | 0.01 |

* Na: Sodium, K: Potassium, CL: Chloride, TP: Total Protein, ALB: Albumin, BUN: Blood Urea Nitrogen, CRE: Creatinine, GLU: Glucose TBIL: Total Bilirubin, Ca: Calcium, PHOS: phosphorous, TCHOL: Total Cholesterol, TG: Triglyceride, AST: Aspartate aminotransferase, ALT: Alanine aminotransferase, CRP: C-reactive protein
